# Microbial community dynamics and bioremediation strategies for petroleum contamination in an in-service oil Depot, middle-lower Yellow River Basin

**DOI:** 10.3389/fmicb.2025.1544233

**Published:** 2025-04-02

**Authors:** Quanwei Song, Bingyu Zhou, Yinan Song, Xianyuan Du, Hongkun Chen, Rui Zuo, Jin Zheng, Tingyu Yang, Yimin Sang, Jufeng Li

**Affiliations:** ^1^CNPC Research Institute of Safety and Environment Technology, Beijing, China; ^2^Department of Environmental Engineering, Beijing Institute of Petrochemical Technology, Beijing, China; ^3^College of Water Sciences, Beijing Normal University, Beijing, China

**Keywords:** in-service oil depot, petroleum hydrocarbon contamination, microbial community, *in-situ* bioremediation, risk management

## Abstract

This study investigated soil and groundwater contamination at an in-service oil transportation station in the middle-lower Yellow River Basin, China. Spatial analysis combined with 16S rRNA and ITS sequencing revealed localized heavy metal (Cu, Ni, Cd, Pb) and petroleum hydrocarbon (PHs: 15.0 mg/kg) contamination in the oily sewage treatment area, with vertical migration constrained by silty sand layers. Volatile organic compounds (VOCs) primarily originated from oil tank emissions. Groundwater exhibited hydraulic gradient-driven downstream migration of PHs (0.03–0.04 mg/L) and arsenic (1.1–1.5 μg/L). Indigenous microbial communities exhibited redox-stratified functional differentiation: unclassified Comamonadaceae (Proteobacteria) dominated aerobic zones (monitoring well D5), utilizing nitrate for PHs degradation, while Desulfosporosinus (Firmicutes) mediated sulfate-coupled anaerobic alkane degradation and metal immobilization in anoxic zones (D6). Fungal communities featured Trametes (Basidiomycota), facilitating ligninolytic PAH breakdown via peroxidase secretion. Functional prediction (FAPROTAX/FUNGuild) confirmed a synergistic “fungal preprocessing-bacterial mineralization” mechanism. Microbial metabolic plasticity (e.g., nitrogen respiration, photoautotrophy) enabled adaptation to redox fluctuations. Given the site’s medium-low risk profile, we proposed a tiered management framework: (1) *in situ* bioremediation that prioritizes indigenous microbes, (2) hierarchical risk zoning, and (3) dynamic monitoring networks. These strategies align with China’s Green Low-Carbon Remediation principles through low-energy microbial technologies. The findings provide a mechanistic basis for balancing industrial operations and ecological health in the Yellow River Basin.

## 1 Introduction

Soil contamination in China has significantly impacted soil quality and land use security (Guan et al., 2020). High-intensity industrial and mining activities are considered the primary sources of soil contamination in China, and several studies have highlighted severe soil contamination and associated risk in regions experiencing rapid industrial development (Lin et al., 2018; Wu et al., 2015; Xu et al., 2014; Yang et al., 2018). Moreover, when soil quality in China is assessed by the level of soil contamination concentration, it is evident that industrial and mining in-service enterprise sites pose more significant threats compared to industrial waste land (Guan et al., 2017). To combat this issue, China has implemented a series of laws and regulations (Li et al., 2019), aiming to standardize the environmental management of enterprises in service and ensure the safety of soil and land resources.

In addition, relevant domestic and international experience indicates that the cost input ratio of end treatment, source prevention, and risk control measures in the remediation of contaminated sites is approximately 100:1:10. The “Action Plan for Ecological Protection and Control of the Yellow River,” released in August 2022, emphasizes focusing on the mainstream and major tributaries of the Yellow River. It requires strict control of the environmental risk from in-service enterprises in industries such as petrochemicals. The contaminated sites from oil depots of in-service petrochemical enterprises are characterized by multiple sources, composite contaminants, coexistence of stock and increment, and high environmental risk (Charmondusit and Keartpakpraek, 2011; Xie et al., 2019; Wang et al., 2020). Once contamination occurs, it is challenging to manage, and thus the remediation cost is high (Elleuch et al., 2018). Nevertheless, historically, China’s industrial land contamination prevention and control efforts have primarily focused on closed industrial contaminated sites (Ma et al., 2023; Xue et al., 2023; Li et al., 2023; Sun et al., 2024). It is evident that this “end treatment” approach is insufficient to address new contamination issues. Currently, soil contamination prevention and control in China is entering a new stage, with the green transformation of the economy and society necessitating the maximum reduction of “incremental” contamination. Therefore, it is imperative to investigate the soil and groundwater of in-service enterprises, identify site contamination, and implement source control measures.

Global oil consumption is increasing annually (Bharti et al., 2021), with China being one of the top countries worldwide in terms of oil refining and consumption (Al-Fattah, 2021). Oil leaks inevitably occur during processing, storage, and use, causing severe contamination to the local or surrounding soil of in-service enterprises such as oil depots, refining and chemical enterprises, and gas stations (Gao et al., 2022; Liu et al., 2015; Wu et al., 2022). Petroleum hydrocarbons are typical contaminants in petroleum-contaminated soil, and selecting appropriate remediation technology is crucial. *In situ* bioremediation is a viable option for contaminated sites of in-service enterprises, as it allows for in-depth remediation without excavation (Kuppusamy et al., 2016), thus avoiding disruption to normal production. *In situ* bioremediation technology has been successfully applied in numerous petroleum-contaminated sites. Numerous robust practical cases demonstrate that microorganisms can efficiently degrade petroleum hydrocarbons by coordinating their metabolic pathways (Huesemann and Moore, 1993; Li et al., 1995; Liebeg and Cutright, 1999). For instance, at a petrochemical site in North China, after 4 years of natural attenuation, concentrations of C10-C40 hydrocarbons decreased by over 70%, with a remarkably short half-life of 693 days (Song et al., 2023). In a fractured karst aquifer in Zibo, Shandong, microbial communities achieved complete mineralization of petroleum hydrocarbons (initial concentration: 161.13 μg/L) via anaerobic respiration over 30 years of natural attenuation, exhibiting a degradation rate of 3.76 × 10^–3^/day. This outcome underscores the long-term efficacy of ecological self-remediation (Guo et al., 2020). Even under highly complex hydrological conditions, natural attenuation effectively curbs contamination spread. For example, at a historical contaminated site in Australia, microbial degradation confined the petroleum hydrocarbons plume to a stable range of 170 meters. Geochemical parameters and carbon isotopic evidence collectively validated the significant role of natural attenuation in blocking contamination diffusion (Naidu et al., 2012). Furthermore, bioaugmentation technology can optimize *in situ* bioremediation. At a contaminated site in Jilin, rational regulation of indigenous microbial communities markedly enhanced petroleum hydrocarbons degradation efficiency (Lv et al., 2018). In a refinery remediation case, targeted addition of functional microbial communities and nutrients increased removal rates of total petroleum hydrocarbons (TPH) and polycyclic aromatic hydrocarbons (PAHs) to 52% and 87%, respectively (Zeneli et al., 2019). In summary, *in situ* bioremediation technology leverages its flexibility to achieve long-term contamination control through natural metabolic networks, while low-disturbance bioaugmentation accelerates remediation. Ultimately, this approach synergizes contamination degradation with ecological restoration, fulfilling the ideal objectives of environmental remediation.

Furthermore, considering that petroleum contaminants can be naturally attenuated through mechanisms such as volatilization, physicochemical adsorption, and indigenous biodegradation (Agnello et al., 2016; Balseiro-Romero et al., 2018), which are highly related to soil physicochemical and biological characteristics (Gogoi et al., 2003; Hamamura et al., 2006; Šolević et al., 2011; Yeom and Ghosh, 1998). Therefore, gaining an in-depth understanding of the distribution and composition of petroleum contaminants, as well as the structure and functional traits of indigenous microbial communities, is of paramount importance for effectively advancing environmental management strategies and ecological remediation efforts at contaminated sites from oil depots.

This study focuses on the contaminated site from an in-service oil depot in central China. By analyzing spatial contamination patterns in soil and groundwater combined with microbial diversity detection, we aimed to: (1) delineate contaminant distribution and identify sources; (2) characterize indigenous microbial communities and assess their functional roles through metabolic pathway predictions (e.g., FAPROTAX/FUNGuild); and (3) establish risk control frameworks and remediation strategies for oil depots in service.

## 2 Materials and methods

### 2.1 Site description

**FIGURE 1:**
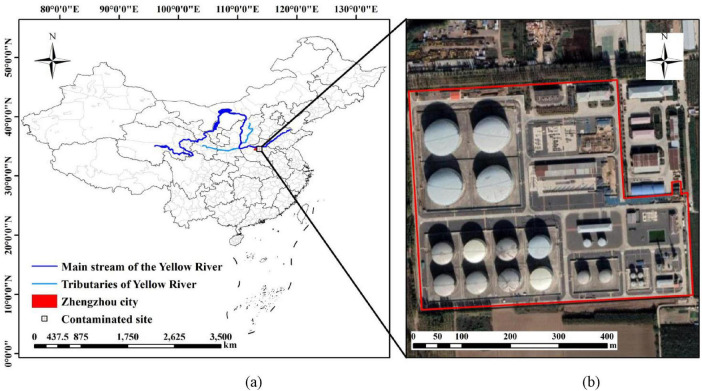
Site conditions. **(a)** Location of contaminated site in the middle-lower Yellow River Basin; **(b)** Satellite schematic map of the investigation scope of oil depot site.

The site of the in-service oil transportation station is located in the central part of Henan Province, which belongs to the middle-lower Yellow River Basin, covering an area of 183,000 m^2^. It belongs to the intermediate station of product oil pipeline transportation. It is engaged in the transportation business of oil products, and the main materials are diesel fuel and gasoline. Silt is commonly found from the surface of the site to the depth of 4.5 m underground, and silty sand is found at 4.5 m to 6 m underground. The shallow groundwater level at the plant site is 12.31 m to 12.59 m, and the groundwater flows from northwest to southeast as a whole. The detailed facility layout of the site is given in Supplementary Material 1.1. The investigation scope of this site is shown in Figure 1.

### 2.2 Sample collection

According to the suspected contaminated areas designation inventory given in the “Technical Provisions for the Layout of Suspected Polluted Plots in Land Use Investigations for Key Industry Enterprises (Trial)” and the key focus areas of in-service enterprises, the suspected contaminated areas of this site were identified. Through the screening and layout principles, the final distribution areas were determined as: 20,000 m^3^ storage tank area, 50,000 m^3^ storage tank area, mixed oil treatment area and oily sewage treatment area. The identification of suspected contaminated areas and confirmation of distribution areas are detailed in Supplementary Material 1.2.

**FIGURE 2:**
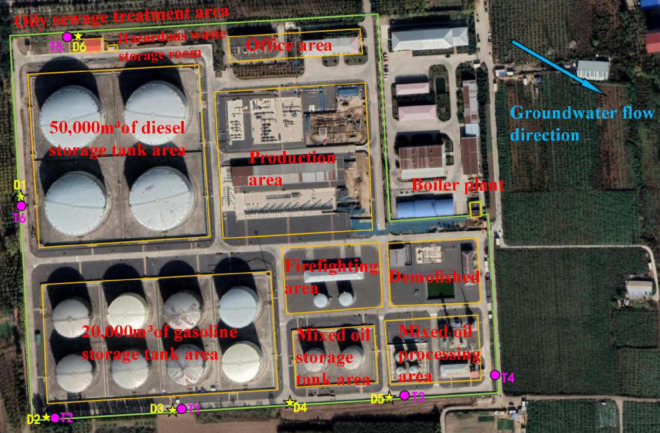
Schematic diagram of soil and groundwater monitoring sites.

Soil sampling strictly followed the “Technical Specifications for Soil Environmental Monitoring” (HJ/T 166-2004). A total of 18 soil samples were collected from six sampling points (excluding two parallel samples) distributed in unhardened areas adjacent to the oil storage tank area, including the southern and southwestern sides of the 20,000 m^3^ tank area, the southwest and southeast of the mixed oil treatment area, the northwest of the oily sewage treatment area, and the western side of the 50,000 m^3^ tank area (Figure 2). At least two sampling points were allocated per sub-area based on contaminant distribution and spatial characteristics. Core samples were collected continuously using stainless-steel augers to avoid cross-layer contamination. Due to the large groundwater depth and absence of visible contamination, soil samples were stratified into three depths: 0 m–0.5 m (surface), 0.5 m–3 m (mid-layer), and 3 m–6 m (deep layer). For T5 (adjacent to a 6 m-deep oily sewage treatment pool), sampling depths were adjusted to 0 m–0.5 m, 0.5 m–3 m, and 3 m–7 m. VOCs samples (≥ 5 g intact cores) were transferred to pre-purged (N_2_) amber vials using sterile syringes (headspace-free), while SVOCs and heavy metals were packed into amber wide-mouth bottles with bamboo spatulas. All samples were labeled with collection time, depth, and ID, stored at 4°C in light-proof containers, and analyzed within 24 h. Quality control included field blanks (pure quartz sand, 1 per 10 samples) and ≥ 10% duplicates.

Groundwater sampling adhered to the “Technical Specifications for Groundwater Environmental Monitoring” (HJ 164-2020) and the “Technical Guidelines for Sampling Volatile Organic Compounds in Soil and Groundwater” (HJ 1109-2019). Six samples (excluding one parallel samples) were collected from existing monitoring wells (depth ≈15 m, water table ≈12.31 m–12.59 m) located in the southeast, south, west, and northwest of the plant (Figure 2). Prior to sampling, 3–5 well volumes were purged using bailers until water quality stabilized (conductivity fluctuation < 10%). Samples were collected 0.5 m below the groundwater surface. VOCs/SVOCs were filled into 40 mL amber vials containing HCl, heavy metals were filtered through 0.45 μm membranes and acidified to pH < 2. All samples were sealed, labeled with well ID and parameters. Part of the samples were stored at 4°C for contamination analysis, and the other part was stored at −*80*°C for biological analysis. Soil and groundwater detection indicators are listed in Supplementary Table 1.

### 2.3 Physicochemical analysis

Environmental contaminant analyses of soil and groundwater samples were performed according to the “Soil Environmental Quality-Risk Control Standard for Soil Contamination of Development Land” (GB 36600-2018) and the “Standard for Groundwater Quality” (GB/T14848-2017). The oil transportation station is mainly engaged in the transportation of oil products, and the land belongs to the category II land of “Soil environmental quality-Risk control standard for soil contamination of development land” (GB 36600-2018). Thus, Category II land risk screening value was selected as the evaluation standard for soil environment investigation. This groundwater evaluation standard was selected the Class III water quality standard of “Standard for Groundwater Quality” (GB/T14848-2017) for evaluation. The indicators not mentioned in the standard, methyl tert-butyl ether and petroleum hydrocarbons (C6-C9 and C10-C40), were evaluated with reference to the “United States. EPA Drinking Water Health Advisories” and the groundwater intervention value of mineral oil in the “Dutch soil and groundwater Environmental Standard” (2013), respectively. The contaminant concentration of all samples are summarized in Supplementary Tables 2 (soil), 3 (groundwater) (Supplementary Table 3).

### 2.4 Illumina sequencing and bioinformatics analysis

Microbial DNA was extracted from groundwater samples (1∼2 L) filtered through 0.22 μm membranes. Initial quality control was performed using SMURF 5R to remove low-complexity sequences. PCR targeted the bacterial 16S rRNA V3–V4 region (primers 338F:5′-ACTCCTACGGGAGGCAGCAG-3′/806R:5′-GGACTACHVGGGTWTCTAAT-3′) and fungal ITS region (primers ITS1F:5′-CTTGGTCATTTAGAGGAAGTAA-3′/ITS2R:5′-GCTGCGTTCTTCATCGATGC-3′). Purified amplicons were sequenced on the Illumina MiSeq PE300 platform (Shanghai Majorbio BioPharm Technology). Raw reads were processed via fastp (v0.19.6) for secondary trimming, FLASH (v1.2.7) for read merging, and DADA2 (Callahan et al., 2016) for amplicon sequence variant (ASV) inference. Taxonomic classification was conducted using QIIME2 (v2022.2) (Bolyen et al., 2019) with classify-sklearn (Naive Bayes), referencing SILVA 138.1 (16S) and UNITE 9.0 (ITS) databases (Quast et al., 2013). Function prediction was subsequently performed, with bacterial ecological roles predicted by FAPROTAX and fungal functional guilds annotated via FUNGuild. Sequencing statistics (raw/high-quality reads, ASV counts) are summarized in Supplementary Table 4, with the procedures of Illumina sequencing detailed in Supplementary Material 1.3.

### 2.5 Statistical analysis

Excel 2019 was used for descriptive statistics, ArcGIS 10.8 was used to map locations, and Origin 2021 was used to analyze and plot data for other figures.

## 3 Results and discussion

### 3.1 Contamination status and spatial distribution of soil and groundwater

#### 3.1.1 Soil contamination and spatial distribution

(1) Soil contamination status

In this investigation, six soil sampling points were set up and 18 normal soil samples were collected. All the soil samples were tested for 47 indexes, namely 45 basic items, pH value and petroleum hydrocarbons (C_10_∼C_40_). Among them, hexavalent chromium, volatile organic compounds (except chloroform, chloroform, dichloromethane, tetrachloroethylene) and semi-volatile organic compounds were not detected in 45 basic projects, the detection rates of arsenic, cadmium, copper, lead, mercury, nickel and methyl chloride were all 100%, the detection rate of chloroform was 83.3%, the detection rate of dichloromethane was 33.3%, the detection rate of tetrachloroethylene was 33.3%, and the detection rate of petroleum hydrocarbons was 5.56%. The 45 basic items and petroleum hydrocarbons in the soil samples are lower than the screening values of the second kind of land in the “Soil Environmental Quality Construction Land Risk Control Standard (trial)” (GB 36600-2018). The overall pH of the site was between 7.99 and 8.65, and the range was small, so the site can be classified as alkaline soil.

(2) Horizontal distribution of soil contamination

1) Heavy metals

**FIGURE 3:**
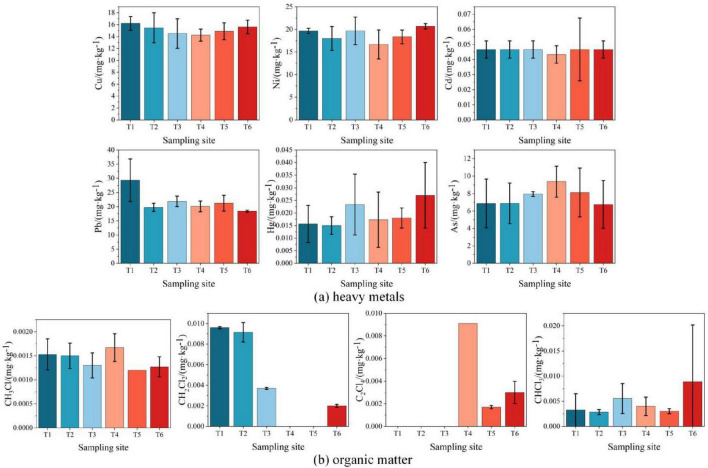
Horizontal distribution of soil contaminants at sampling points T1–T6. **(a)** Heavy metals; **(b)** Organic matter.

Six heavy metals (copper, nickel, cadmium, lead, mercury, and arsenic) were detected at all sampling points (T1–T6), with concentrations significantly lower than the category I and II land risk screening values in GB 36600-2018 (Figure 3a). Notably, lead concentrations at T1 (south of the 20,000 m^3^ gasoline storage area) were markedly higher than at other points. This anomaly was attributed to non-stationary anthropogenic sources, as no evidence of equipment leakage or historical contamination was identified.

2) Organic contaminants

The organic contaminants at T1∼T6 points was detected, among which petroleum hydrocarbons were only detected at T5 (15.0 mg/kg), and other organic contaminants were detected as shown in Figure 3b. Chloromethane, dichloromethane, tetrachloroethylene, and chloroform were detected at trace levels (0.01%–1% of the category I screening values in GB 36600-2018). Overall, the level of soil contamination is low. A comprehensive analysis of soil contamination horizontal distributions is provided in Supplementary Material 2.1.

(3) Vertical distribution of soil contamination

1) pH value

Soil pH value is one of the important indicators to reflect the physical and chemical properties of soil, which not only controls the existing form and bioavailability of metal elements and many other physical and chemical processes (Xu et al., 2020), but also has an important impact on the activities of bacteria and fungi in soil (Aziz et al., 2024). Therefore, exploring the spatial distribution of soil pH is helpful to clarify the migration process of contaminants in the site, and can provide a basis for the use of soil microorganisms for risk control of remediation.

**FIGURE 4:**
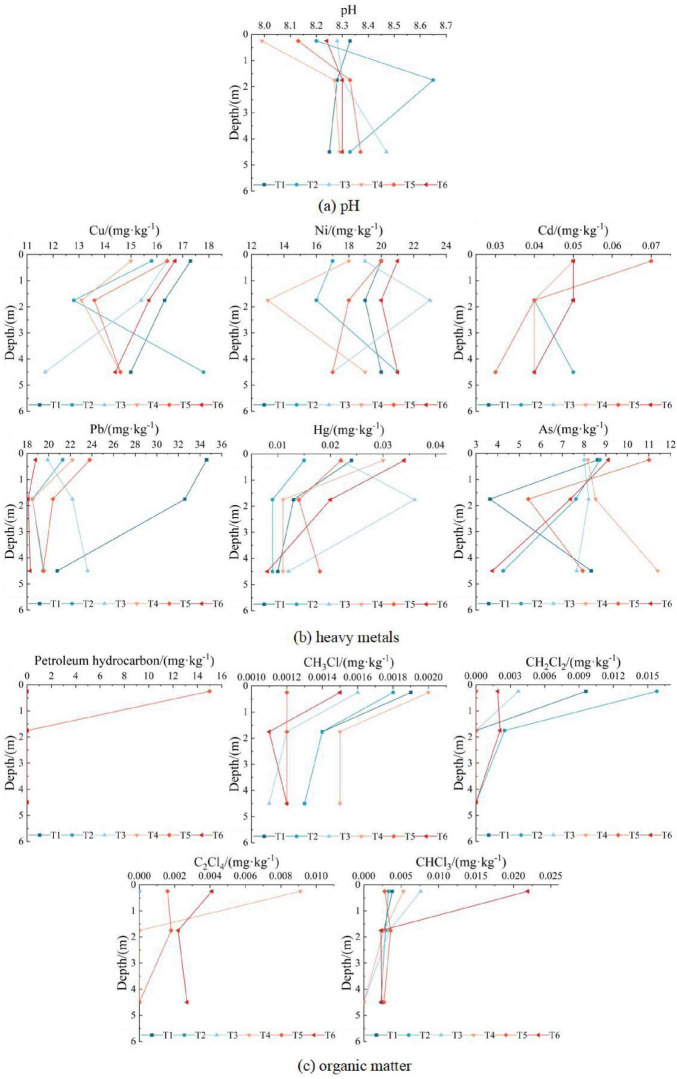
Vertical distribution of soil contaminants at sampling points T1–T6. **(a)** pH; **(b)** Heavy metals; **(c)** Organic matter.

Soil pH increased significantly from the surface (0 m–0.5 m) to the middle layer (0.5 m–3.0 m), stabilizing at approximately 8.3 in deeper soil (3.0 m–6.0 m) (Figure 4a). This trend reflects enhanced alkalinity with depth, likely influenced by soil texture and microbial activity.

2) Heavy metals

The vertical distribution of heavy metals (Figure 4b) reveals a predominant “surface enrichment” pattern, where concentrations of most metals (Cu, Ni, Cd, Pb) peak in topsoil (0 m–3 m). This trend correlates with the finer texture (silt, the content of particles larger than 0.075 mm is not more than 50% of total weight) and higher organic matter content in surface soil, which enhance metal retention through adsorption and humus complexation (Yang et al., 2013). In contrast, deeper layers (3 m–6 m) exhibit coarser particles (silty sand, the content of particles larger than 0.075 mm is more than 50% of total weight) and reduced organic matter, weakening metal immobilization. Notably, however, several metals deviate from this generalized pattern due to point-specific contamination pathways and hydrogeochemical controls.

Beyond these overarching trends, four distinct contamination pathways dominate: At T2/T4/T5, Cu/Ni/Hg/As concentrations decrease then increase with depth (even exceeding surface levels), attributed to historical fertilizer residues migrating downward via rainfall leaching; At T3, Ni/Hg peak at 0.5 m–3 m due to invalid anti-seepage infrastructure, with low permeability in middle layers trapping contaminants; Pb at T3 and As at T4 gradually rise with depth, linked to legacy pesticide/fertilizer transport through rainwater infiltration; At T1/T2/T4, Hg concentrates in topsoil (0 m–0.5 m), while deeper layers stabilize near regional background levels, indicating minimal contamination penetration.

Analysis of six heavy metals revealed a general downward concentration trend with soil depth, except at specific points where historical land use caused elevated deep-layer contamination. Notably, surface soil at T5 exhibited higher concentrations of all six metals compared to deeper layers. Correlated with exclusive petroleum hydrocarbons detection at T5 (15.0 mg/kg), this indicates operational contamination from oil leakage or waste residue accumulation (e.g., tank sludge, oily rags). Additionally, anomalous nickel and mercury distribution at T3 suggests aging anti-seepage materials.

3) Organic contaminants

The vertical distribution of organic matter is shown in Figure 4c. Petroleum hydrocarbons were detected only in the surface soil at T5 point, and the mass concentrations in the middle and deep layers were lower than the detection limits, which further proved that petroleum hydrocarbons detected at T5 point were caused by oil contamination at the site from oil depot, rather than historical contamination sources. The other 4 organic contaminants were VOCs, exhibiting “surface enrichment,” with concentrations sharply declining from surface to mid-layers (0 m–3 m) and stabilizing at greater depths. At T1–T4 and T6, where chloroform, dichloromethane, or tetrachloroethylene were detected, contaminant concentrations exhibited a characteristic trend of initial decrease followed by stabilization with increasing soil depth. Spatial analysis revealed that VOCs originated primarily from oil tank emissions (T1/T2: 20,000 m^3^ tank area; T6: 50,000 m^3^ tank area) and heating furnace flue gas emissions (T3/T4: mixed oil treatment area). These VOCs volatilized into the atmosphere, adsorbed onto particulates, and deposited onto surface soil via dust precipitation. Chloroform distribution at T1–T4 was uniform across depths, indicating no operational contamination, and its presence, which was likely derived from natural sources, further proves the previous speculation. In contrast, T6 displayed surface enrichment of chloroform, likely due to oil tank breathing; At T5 (northwest corner), northwest winds during sampling minimized atmospheric contamination, resulting in stable vertical distributions of chlorinated VOCs. A comprehensive analysis of soil contamination vertical distributions is provided in Supplementary Material 2.2.

#### 3.1.2 Groundwater contamination and spatial distribution

(1) Groundwater contamination status

Odor and taste, visible to the naked eye, copper, zinc, anionic surfactant, sulfide, total coliform, cyanide, iodide, mercury, selenium, cadmium, lead, carbon tetrachloride, benzene, toluene, ethylbenzene, total xylene (o-xylene + m/p-xylene) and methyl tert-butyl ether were not detected in groundwater samples. The detected values of color, turbidity, pH, total hardness, total dissolved solids, sulfate, chloride, iron, manganese, aluminum, volatile phenols, oxygen consumption, ammonia nitrogen, sodium, total bacterial count, nitrate, fluoride, arsenic, chromium (hexavalent), trichloromethane, total α radioactivity and total β radioactivity all meet the requirements of Class III in the “Standard for groundwater quality” (GB/T14848-2017). The total petroleum hydrocarbons (C_6_-C_9_ + C_10_-C_40_) index meets the groundwater intervention value requirements of mineral oil in the “Dutch soil and groundwater Environmental Standard” (2013).

(2) Horizontal distribution of groundwater contamination

1) Heavy metals

**FIGURE 5:**
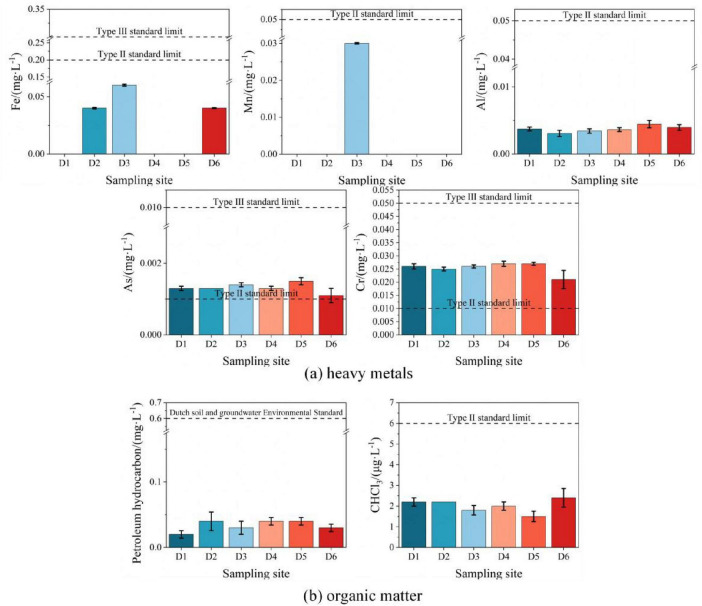
Horizontal distribution of groundwater contaminants in D1–D6 monitoring wells. **(a)** Heavy metals; **(b)** Organic matter.

Among the five detected heavy metals, the concentrations of iron, manganese, and aluminum in all monitoring wells were below the Class II standard limits of GB/T14848-2017 (Figure 5a); however, arsenic and chromium concentrations in wells D1–D6 exceeded the Class II groundwater standards. Specifically, D6 (upstream) exhibited significantly lower concentrations of arsenic and chromium compared to D5 (downstream). This spatial distribution aligns with the groundwater flow direction (northwest to southeast), indicating contaminant dispersion from upstream to downstream.

2) Organic contaminants

Petroleum hydrocarbons were detected in all monitoring wells (D1–D6), but soil contamination was only observed at T5 (D6 area, oily wastewater treatment area) (Figure 5b). Groundwater flow analysis indicates D6 contamination originated from soil leaching due to oil sewage leaks or residue accumulation at T5, while other wells (D1–D5) were contaminated via downstream groundwater transport. Notably, D6 exhibited lower concentrations than downstream D5, consistent with arsenic/chromium distribution, suggesting contaminant enrichment through groundwater migration. Chloroform levels in all wells were uniformly low (below Class II/III standards) and likely derived from natural soil leaching (high mobility under rainwater infiltration), rather than direct human activities (Ya-song et al., 2011). A comprehensive analysis of groundwater contamination horizontal distributions is provided in Supplementary Material 2.3.

#### 3.1.3 Total contamination and its risk

Monitoring data indicate that the contamination characteristics of soil and groundwater in the study area exhibit significant spatial heterogeneity. Specifically, at the T5 point, there is an exhibition of a “surface enrichment” phenomenon of petroleum hydrocarbons (15.0 mg/kg) and multiple heavy metals (Cu, Ni, Cd, Pb), with their vertical migration constrained by silty sand layers. Groundwater contamination is characterized by dissolved-phase diffusion, with slightly higher concentrations of petroleum hydrocarbons (0.04 mg/L) and arsenic (1.5 μg/L) in the downstream monitoring well D5 compared to the upstream well D6 (0.03 mg/L, 1.1 μg/L). This slight enrichment of contaminants along the northwest to southeast groundwater flow direction aligns with the hydraulic gradient-driven pattern. Despite localized contamination hotspots, all soil and groundwater indicators are below the “risk screening values for contamination of land for construction.” According to the definition of “risk screening value” in the “Terms of risk control and remediation of soil contamination of land for construction” (HJ682-2019), it can be concluded that the risk of pollutants in the soil and groundwater of the site to human health is negligible. Therefore, the site can be considered to be in the medium-low risk category.

Based on the aforementioned risk characteristics, a “natural attenuation” remediation approach is recommended. Notably, the spatial distribution of contaminants in the study area (e.g., petroleum hydrocarbons enrichment at T5, heavy metal gradients at D5/D6) is significantly correlated with the redox conditions of groundwater. This environmental heterogeneity provides a key driving force for the functional differentiation of indigenous microorganisms. The nitrate concentration at D5 (13.4 mg/L) is significantly higher than at D6 (6.81 mg/L), and although the sulfate concentration at D5 is higher (83.2 mg/L vs. 66.8 mg/L at D6), the reduction is only 19.7%, indicating that the oxygen-rich environment may inhibit the activity of sulfate-reducing bacteria. In contrast, the D6 monitoring point, with undetectable dissolved oxygen, is in an anoxic state, in which nitrate consumption and slight sulfate reduction indirectly suggest that sulfate is acting as a secondary electron acceptor in anaerobic metabolic processes. The co-evolution of geochemical differentiation and microbial community metabolic functions manifests as a typical functional partitioning in such remediation mechanisms. Specifically, aerobic hydrocarbon-degrading bacteria (e.g., Proteobacteria) in the oxygen-rich and nitrate-abundant D5 region efficiently mineralize C10–C40 long-chain hydrocarbons enriched at the T5 surface, while sulfate-reducing bacteria (e.g., Firmicutes) in the low-oxygen environment of D6 immobilize dissolved arsenic and chromium through sulfide generation pathways. This self-regulating pattern aligns with internationally studied microbial remediation pathways (Brassington et al., 2007; Sui et al., 2021; Van Stempvoort and Biggar, 2008; Wang et al., 2022), but its applicability to medium-low risk contaminated sites of in-service oil depots in China requires further validation.

### 3.2 Indigenous microbial communities and *in situ* bioremediation feasibility

#### 3.2.1 Indigenous microbial communities

The microbial sampling points D5 and D6 were selected based on the documented contamination heterogeneity and redox stratification (see section “3.1 Contamination status and spatial distribution of soil and groundwater”). D6, situated in the T5 source area, exhibited hydrocarbon enrichment with co-occurring heavy metal accumulation in surface soils, while the downstream site D5 showed contaminant redistribution through hydrogeochemical transport. Pronounced redox contrasts were observed: D6 maintained an anoxic, nitrate-depleted microenvironment, whereas D5 supported oxic conditions with available nitrate and sulfate. This geochemical gradient enabled systematic investigation of microbial adaptive strategies to hydrocarbon stress. Bacterial (BD5, BD6) and fungal (FD5, FD6) communities were analyzed to determine three critical functional attributes of indigenous consortia: (1) intrinsic degradation capacity, (2) environmental adaptability under redox fluctuations, and (3) interdomain functional complementarity (B: bacteria; F: fungi). These attributes collectively validate the implementation of *in situ* bioremediation leveraging indigenous microbial ecology for sustainable risk management.

(1) Sequencing quality evaluation

**FIGURE 6:**
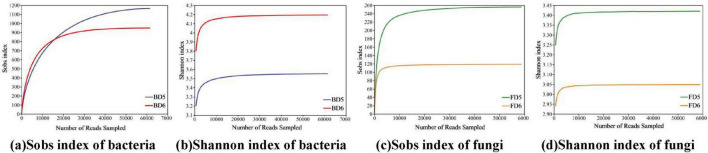
Sobs and Shannon index rarefaction curves of microbial communities. **(a)** Sobs index of bacteria; **(b)** Shannon index of bacteria; **(c)** Sobs index of fungi; **(d)** Shannon index of fungi.

We analyzed the microbial community composition using bacterial (BD5, BD6) and fungal (FD5, FD6) samples obtained from contaminated groundwater. After rigorous quality filtering, high-quality reads for bacterial samples ranged from 61,587 to 64,472 (BD5–BD6), while fungal samples retained 58,913 to 97,819 reads (FD5–FD6) (Supplementary Table 4), demonstrating sufficient sequencing depth. Rarefaction curves for observed species richness (Sobs) and Shannon diversity index (Figure 6) plateaued at the current sequencing depth, confirming comprehensive coverage of microbial diversity.

(2) Alpha diversity of bacterial and fungal communities

The Alpha diversity index was used to quantitatively describe the diversity of bacteria and fungi in the groundwater at points D5 and D6, as shown in Table 1.

**TABLE 1:** Alpha diversity index of microorganisms in different groundwater samples.

Sample	Community richness			Community diversity		Community evenness	Community coverage
	Sobs	Chao	ACE	Shannon	Simpson	Pielou_e	Coverage
BD5	1,166	1167.50519	1175.930145	3.553198	0.129141	0.503190731	0.999513
BD6	949	949	949	4.194382	0.054145	0.611835431	1
FD5	256	256	256.246547	3.419829	0.077189	0.638013017	0.999983
FD6	119	119	119	3.049143	0.115316	0.616721292	1

The diversity index serves as a comprehensive tool to evaluate the richness and evenness of microbial communities. For the bacterial community, although BD5 has higher richness indices (Sobs, Chao, ACE) than BD6, BD6 exhibits a higher Shannon Index. This suggests that while BD5 contains more species, the species abundance distribution in BD6 is more even, leading to a higher overall diversity. For the fungal community, the combined richness and evenness indexes indicate that FD5 has a higher diversity than FD6. It is evident that the diversity of microorganisms varies across different groundwater monitoring wells, which aligns with the conclusion that the concentration of petroleum hydrocarbons and characteristic factors of the groundwater environment can significantly influence the diversity of microbial communities (Ning et al., 2018). Additionally, the Coverage index for all four samples was above 99%, indicating that the sequences obtained through sequencing essentially included all sequences present in the samples, and thus the sequencing results accurately represent the actual distribution of bacteria and fungi in the samples.

(3) Composition of bacterial and fungal communities

1) Venn diagram

Firstly, the ASV quantities of 4 groundwater samples at each classification level were shown, as shown in Table 2.

**TABLE 2:** Amplicon sequence variant (ASV) species classification statistics of microorganisms in different groundwater samples.

Samples	Phylum	Class	Order	Family	Genus
BD5	40	98	196	274	374
BD6	29	79	168	249	409
FD5	6	22	50	83	116
FD6	8	17	29	42	54

**FIGURE 7:**
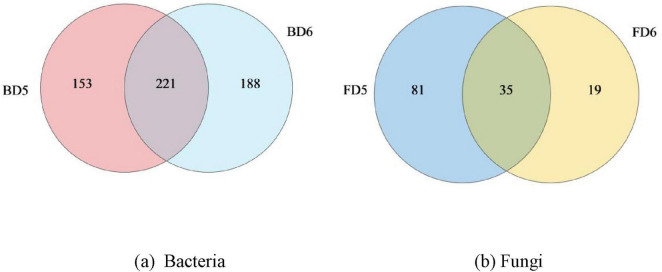
Differences in the composition of bacteria and fungi at the genus-level in different groundwater samples. **(a)** Bacteria; **(b)** Fungi.

Secondly, Venn diagram was used to show the number of shared and unique bacterial and fungal genera between BD5 and BD6, FD5 and FD6. As can be seen from Figure 7a, 374 bacterial genera were detected in BD5, of which 153 were unique and only 221 were shared with BD6. As can be seen from Figure 7b, 116 fungal genera were detected by FD5, while 81 fungal genera were unique and only 35 fungal genera were shared, even less than those unique to D5, indicating that the species composition of samples from D5 and D6 groundwater monitoring wells was significantly different.

2) Bar diagram and Circos diagram

**FIGURE 8:**
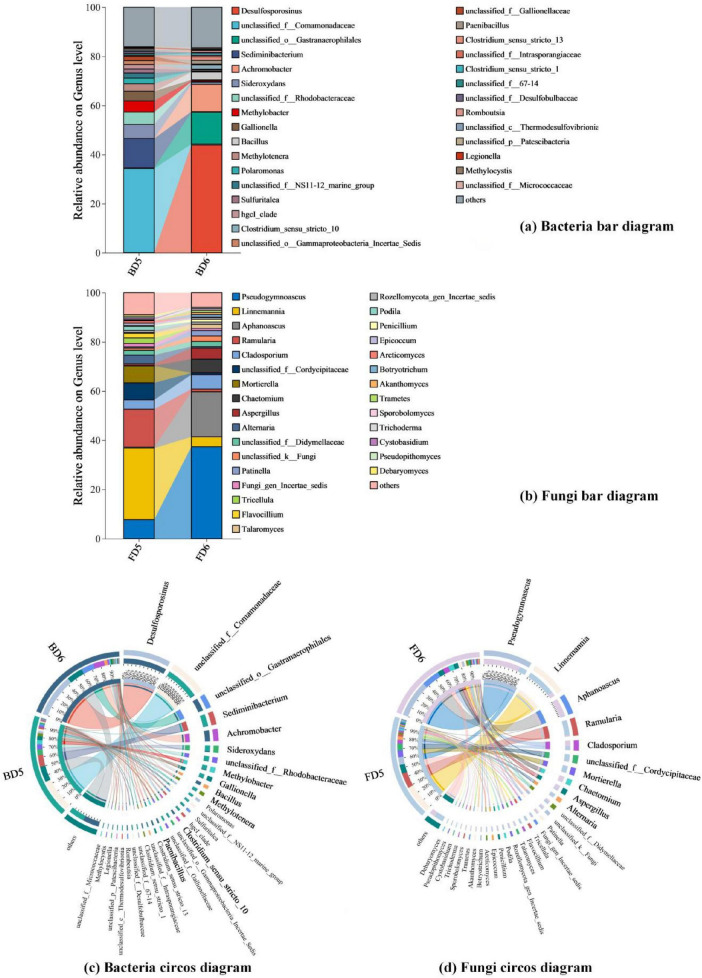
Composition of bacterial and fungal genera present in different groundwater samples. **(a)** Bacteria bar diagram; **(b)** Fungi bar diagram; **(c)** Bacteria circos diagram; **(d)** Fungi circos diagram.

Bar diagrams and Circos diagrams (genus level: Figure 8; phylum level: Supplementary Figures 1, 2) were generated to visualize both the compositional structure of microbial communities and the relative abundances of key taxonomic groups within each sample.

For bacterial community, BD5 at the phylum level was dominated by Proteobacteria (70%), followed by Bacteroidota (15%), Actinobacteriota (4%), Patescibacteria (3%), and Desulfobacterota (2%). The dominance of Proteobacteria was primarily attributed to unclassified Comamonadaceae (34%) and Methylobacter (5%). Members of unclassified Comamonadaceae facilitate aromatic hydrocarbon co-metabolism through hybrid metabolic pathways (Jiang et al., 2023), while Methylobacter employs methane monooxygenase to oxidize short-chain alkanes (Bowman, 2006). Notably, Sideroxydans (6%, Proteobacteria), a genus specialized in iron oxidation, further supports Fe-coupled aerobic hydrocarbon degradation (Zhou et al., 2022). In contrast, BD6 exhibited a distinct bacterial composition dominated by Firmicutes (61%), with secondary contributions from Proteobacteria (18%), Cyanobacteria (13%), Actinobacteriota (3%), and Bacteroidota (2%). Within Firmicutes, Desulfosporosinus (44%) emerged as the key genus, coupling long-chain alkane degradation to dissimilatory sulfate reduction under anaerobic conditions (Agrawal and Gieg, 2013). The cumulative abundances of hydrocarbon-degrading bacterial phyla (Proteobacteria (Fuentes et al., 2014; Lasher et al., 2009; Shahi et al., 2016; Sun et al., 2015), Firmicutes (Yang et al., 2014; Wang et al., 2019), Bacteroidota (Chen et al., 2021), Actinobacteriota (Thomas et al., 2019; Baoune et al., 2018), Desulfobacterota) reached 91% in BD5 and 84% in BD6, confirming their robust biodegradation potential. This aligns with previous studies identifying these phyla as critical hydrocarbon degraders.

For fungal communities, FD5 was dominated by Ascomycota (54%) and Mortierellomycota (38%), while Basidiomycota constituted only a minor fraction (5%). Despite their low abundance, Basidiomycota played a disproportionately significant role in hydrocarbon degradation. Key genera within Basidiomycota, including Trametes, contributed to this process by secreting ligninolytic peroxidases, such as lignin peroxidase (Llado et al., 2012). These enzymes catalyze the oxidation of complex aromatic structures, thereby enabling Trametes to degrade recalcitrant polycyclic aromatic hydrocarbons (PAHs), which are among the most hazardous components of petroleum contaminants. In FD6, Ascomycota achieved near-exclusive dominance (87%), primarily driven by Pseudogymnoascus (37%) and Aphanoascus (18%), both of which exhibit hyphal adaptations for penetrating hydrophobic hydrocarbon matrices and enhancing enzymatic hydrolysis under anoxia. Fungi involved in the biodegradation of petroleum hydrocarbons mainly include Ascomycota and Basidiomycota (Ramdass and Rampersad, 2021), and the relative abundance of Ascomycota and Basidiomycota in the fungal communities of groundwater monitoring wells D5 and D6 (FD5 and FD6) is 59% and 91%, respectively, both accounting for a large proportion of the entire fungal community, indicating that the fungal communities in the two monitoring wells also have good ability to degrade petroleum hydrocarbons. Fungi may surpass bacteria in certain niches due to their hyphal mobility, hypoxia tolerance, and capacity to metabolize recalcitrant compounds (Liu et al., 2011).

(4) Functional prediction of bacterial and fungal communities

**FIGURE 9:**
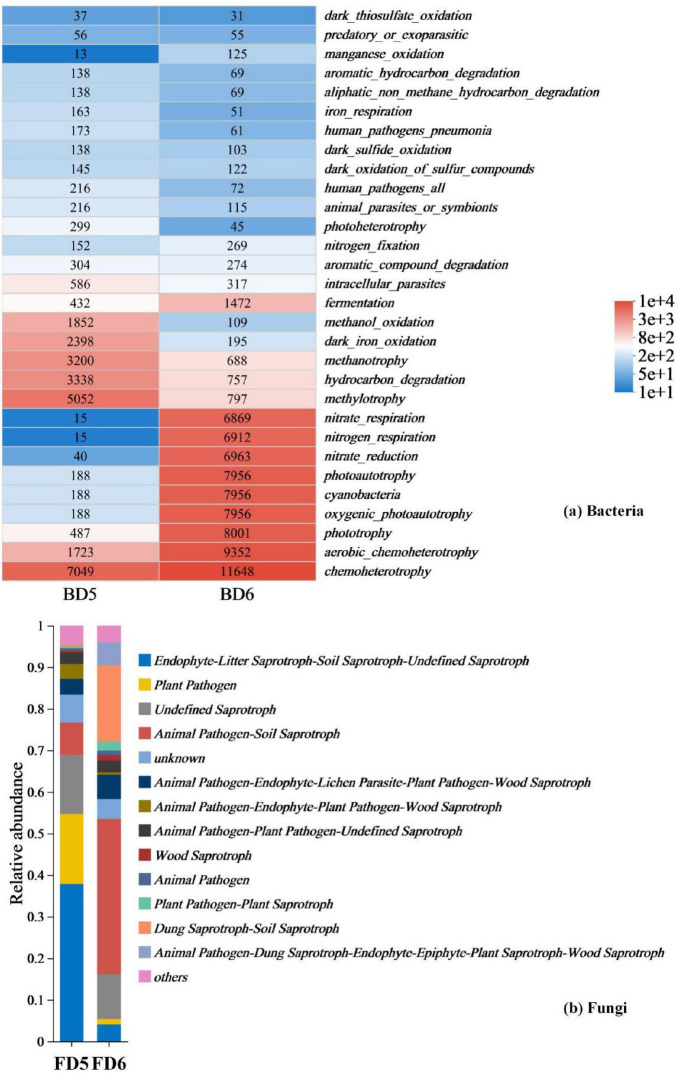
Prediction of bacterial and fungal communities function present in different groundwater samples. **(a)** Bacteria; **(b)** Fungi function prediction.

Functional prediction data from bacterial (BD5, BD6) and fungal (FD5, FD6) communities (fungal abundance data listed in Supplementary Table 5) reveal the *in situ* petroleum degradation potential of indigenous microorganisms (Figure 9). The hydrocarbon degradation activity (hydrocarbon_degradation: 3338) in BD5 is significantly higher than that in BD6 (757), and this difference correlates with its nitrate - rich environment, suggesting functional enrichment of degradation pathways under high contamination conditions. For instance, methylotrophs (methylotrophy: 5,052) can utilize methane and short-chain alkanes as carbon sources, a metabolic flexibility recognized as a key mechanism in oil-contaminated environments (Yakimov et al., 2007).

Fungal functional data further validate the integrity of the degradation chain. The high abundance of undefined saprotrophs (Undefined Saprotroph: 8,362) and wood saprotrophs (Wood Saprotroph: 304) in FD5 suggests their role in cleaving polycyclic aromatic hydrocarbons (PAHs) into smaller molecules via lignin peroxidase secretion (Harms et al., 2011), which are subsequently mineralized by bacteria. This “fungal pre-degradation-bacterial mineralization” division of labor (Boonchan et al., 2000) operates autonomously without exogenous inoculants, emphasizing the self-sufficiency of *in situ* remediation.

The metabolic plasticity of microbial communities is crucial for responding to environmental gradients. The 40-fold increase in oxygenic photoautotrophy (7956) in BD6 compared to that in BD5 (188) demonstrates microbial reliance on photosynthesis and nitrogen respiration (nitrogen_respiration: 6,912) to reconstruct energy acquisition pathways under carbon-limited conditions. Concurrently, the shift in fungal functional guilds from plant pathogens (Plant Pathogen: 9,934) in FD5 to saprotroph-pathogen dual-functional groups in FD6 may maintain ecological stability by degrading residual pollutants (Purahong et al., 2016).

Cross-domain synergy between bacteria and fungi further enhances remediation efficiency. For example, methanotrophs in BD5 depend on fungal-derived methanol (methanol_oxidation: 1,852) for metabolism, while cyanobacteria in BD6 (7,956) release oxygen via photosynthesis, activating aerobic saprotrophs (e.g., Dung Saprotroph-Soil Saprotroph: 10,796) in FD6 to form a redox-regulated degradation network. These findings demonstrate that indigenous microorganisms independently drive petroleum remediation through metabolic division, environmental adaptability, and cross-domain synergy.

Although functional predictions (FAPROTAX/FUNGuild) are inherently theoretical, their ecological relevance is supported by convergent taxonomic and geochemical patterns. For instance, the dominance of unclassified Comamonadaceae in BD5 (34% relative abundance) aligns with their established role in aerobic hydrocarbon degradation via nitrate respiration (Li et al., 2012), while Desulfosporosinus (44% in BD6) corresponds to sulfate-reducing syntrophy in anaerobic oil reservoirs (Agrawal and Gieg, 2013). These taxonomic associations are further reinforced by point-specific geochemical gradients: elevated nitrate in D5 (13.4 mg/L) selects for oxygen-tolerant degraders (FAPROTAX: 3,338 hydrocarbon degradation counts), whereas nitrate depletion in D6 (6.81 mg/L) promotes sulfate-dependent taxa (6,912 nitrogen respiration counts).

#### 3.2.2 *In situ* bioremediation feasibility

Systematic analysis of microbial communities in groundwater samples D5 and D6 robustly supports the feasibility of *in situ* bioremediation at the contaminated site. Despite distinct microbial diversity driven by hydrocarbon concentration and hydrogeochemical gradients, both samples contained metabolically active and environmentally adaptable functional taxa. High-throughput sequencing demonstrated sufficient depth and comprehensive coverage, ensuring reliable characterization of indigenous microbial dynamics. Indigenous microbes exhibited specialized hydrocarbon degradation capacities, including Proteobacteria (BD5: 70%) and Firmicutes (BD6: 61%) dominated bacterial communities, with key genera such as unclassified Comamonadaceae, Sideroxydans, and Desulfosporosinus which degrade hydrocarbons via aerobic oxidation or sulfate-coupled anaerobic pathways. Fungal communities (FD5/FD6) were enriched with Ascomycota and Basidiomycota, among which Trametes participated in complex hydrocarbon degradation through lignin peroxidase secretion and hypoxia-adapted enzymatic hydrolysis. Critically, although direct quantification of functional biomarkers (e.g., alkB, dsrAB) was not performed, our reliance on taxonomically-resolved functional proxies is empirically justified. The absence of direct biomarker quantification (e.g., alkB, dsrAB) is mitigated by taxonomic proxies empirically linked to functional potential. For example, unclassified Comamonadaceae dominance in nitrate-rich D5 mirrors their documented alkB gene abundance in aerobic hydrocarbon plumes (Sun et al., 2015), while Desulfosporosinus (D6) is a recognized sulfate reducer encoding dsrAB in oil-saturated environments (Varon-Lopez et al., 2014). Notably, Trametes fungi (FD5) provide ligninolytic enzymes (LiP, MnP) to oxidize PAHs into bioavailable intermediates, a mechanism validated via transcriptomics in contaminated soils (Harms et al., 2011). These taxonomy-function linkages are well-characterized in oilfield microbiomes (Zeneli et al., 2019), underscoring the rationale for prioritizing indigenous microbial consortia in remediation strategies. Building on these robust taxonomic indicators, functional predictions further revealed microbial self-organization: saprotrophic fungi fragmented PAHs via enzymatic hydrolysis, while bacteria mineralized the byproducts. This interaction formed a synergistic “fungal preprocessing-bacterial mineralization” mechanism. Additionally, microbial communities exhibited remarkable metabolic plasticity, dynamically adapting their energy acquisition strategies (e.g., nitrogen respiration and photoautotrophy) to environmental fluctuations. These traits ensure functional stability under fluctuating redox conditions and closely align with the ecological principles of natural attenuation. In conclusion, the degradation efficiency, environmental adaptability, and cross-domain synergy of indigenous microorganisms collectively validate *in situ* bioremediation. We advocate implementing an *in situ* bioremediation strategy that capitalizes on indigenous microbial communities to degrade contaminants, ultimately establishing an ecologically sustainable risk management paradigm (Lv et al., 2018; Mafiana et al., 2021; Santos Oliveira et al., 2013; Song et al., 2023).

The above findings demonstrate that the metabolic division of indigenous microbial communities is highly compatible with the redox gradients of the contaminated environment. This self-organizing remediation mechanism provides a scientific basis for “*in situ* bioremediation technologies.” However, to ensure remediation efficiency and ecological safety, it is imperative to develop dynamic risk management strategies tailored to area-specific contamination characteristics and validate remediation efficacy through long-term monitoring.

### 3.3 Risk management and control of medium-low risk contaminated sites from in-service oil depots

Focusing on the in-service oil depot as a critical case, this study synthesizes investigation data and indigenous microbial community characteristics to provides countermeasures and suggestions for advancing soil-groundwater risk management and control in contaminated sites.

(1) Investigation on hidden dangers of soil and groundwater contamination

In the normal production and operation of the pipe network oil depot, in order to ensure the continuous and effective prevention of soil and groundwater contamination caused by the leakage, loss and dispersion of toxic and harmful substances in key places or key facilities and equipment, and self-organized investigation of hidden dangers of soil contamination. With reference to “Guidelines for the Investigation of Hidden Dangers of Soil Contamination in Key Supervision Units,” establish a system for the investigation of hidden dangers of soil contamination, find the hidden dangers of soil contamination in time and take measures to eliminate or reduce the hidden dangers.

Regular inspections are carried out for key areas and key facilities in oil depot production activities, such as refined oil or mixed oil storage areas, oily wastewater treatment areas and temporary storage rooms for hazardous wastes. In principle, enterprises take the factory area as a unit to carry out a comprehensive and systematic investigation of soil contamination hidden dangers every 2∼5 years. Enterprises can optimize and adjust the frequency and scope of investigation according to the characteristics of the industry and the reality of production. For places where production processes, facilities and equipment have changed, or newly renovated and expanded areas, supplementary inspection shall be carried out within 1 year. As a key regulatory unit, oil depots may organize and carry out inspection on their own according to their own technical capabilities, or entrust relevant technical units to assist in the investigation.

(2) Self-monitoring of soil and groundwater

In order to strengthen the supervision and management of the environmental protection of soil and groundwater of in-service oil depots, and prevent and control the contamination of soil and groundwater of in-service oil depots, it is recommended to carry out self-monitoring of soil and groundwater of in-service oil depots with reference to the “Technical Guide for Self-Monitoring of Soil and Groundwater of in-service Enterprises.”

In-service oil depots should further optimize the layout of monitoring wells in different types of key areas, such as oil storage tanks, oily wastewater treatment areas, hazardous waste temporary storage rooms and underground oil pipelines, combined with the actual situation on the spot, and take the characteristic contaminants determined by site investigation, such as petroleum hydrocarbons, heavy metals and common petrochemical site characteristic contaminants such as BTEX (Dantas et al., 2022) and PAHs (Al-Hawash et al., 2018; Hu et al., 2010; Benlaribi and Djebbar, 2020; Domínguez-Morueco et al., 2018; Premnath et al., 2021), as site characteristic contaminants to carry out normalized self-monitoring. According to the actual situation, a soil investigation is carried out every 3∼5 years, the soil sampling depth and layers are classified and determined according to different types of key areas, the number of key facilities and the degree of suspected contamination, and groundwater monitoring is carried out once a year. Groundwater samples are selected according to stratigraphic characteristics, hydrogeological characteristics and on-site contamination degree. Through long-term scientific monitoring and tracking, the rolling application and long-term application of the investigation results can be realized, so as to provide a reliable basis for contamination control and prevention.

(3) Remediation of soil and groundwater contamination

The risk management and control of medium-low risk contaminated sites from in-service oil depots face significant challenges, necessitating the dual objectives of ensuring uninterrupted production activities while effectively controlling contaminant dispersion and migration. Consequently, the selection of remediation technologies must adhere to the principle of non-interference with industrial operations, aiming to achieve efficient contaminant degradation and establish sustainable environmental risk management mechanisms. Under China’s policy framework, multiple legislative documents explicitly advocate for the development and implementation of *in situ* remediation technologies that are tailored to local environmental conditions and exhibit minimal ecological footprint. Notably, “The Guidance on Promoting Soil Pollution Risk Management and Green Low-Carbon Remediation” (issued in December 2023) reaffirms that on the basis of paying attention to economic feasibility, it should emphasize the guidance of resource and energy conservation and efficient utilization, optimize process design, and give priority to low disturbance remediation technologies such as *in situ* remediation, bioremediation, and natural recovery.

Aligned with both practical demands and policy orientation, *in situ* bioremediation has garnered substantial attention due to its distinct advantages. *In situ* bioremediation technology leverages microbial metabolic processes to degrade contaminants within soil and groundwater. By eliminating the need for large-scale excavation or structural modifications, this approach minimizes disruptions to production activities. In addition, it has the characteristics of simple operation, low energy consumption, and high remediation efficiency, which synergistically aligns with national strategies for advancing green and low-carbon development agendas. Through long-term on-site monitoring and regular effect evaluation, *in situ* bioremediation technology can effectively inhibit the diffusion process of contaminants, promote the natural attenuation and chemical degradation of contaminants, and thereby achieve the long-term treatment of soil and groundwater contamination as well as risk control objectives. This remediation scheme can not only meets the actual needs of enterprises in service, but also meets the current national policy requirements and technical development trends for soil and groundwater contamination control.

### 3.4 Countermeasures on environmental management of contaminated sites from in-service oil depots

The effective implementation of risk management measures relies on policy support and technological synergy. For instance, the promotion of *in situ* remediation technologies requires institutionalized self-monitoring practices (e.g., annual groundwater sampling), while the long-term stability of microbial remediation efficacy necessitates national standards to regulate technical workflows. The following multidimensional management countermeasures are proposed to ensure the sustainable achievement of risk control and remediation goals.

**(1) At the level of the country, improve the relevant systems to provide protection for the site contamination prevention and control system of petrochemical enterprises**

China’s soil and groundwater environmental management is still in its infancy, the soil contamination prevention and control system of petroleum and petrochemical enterprises needs to be improved, systematic management methods have not yet been formed (Li et al., 2017), and environmental protection managers are lack of soil-related professional knowledge, site contamination prevention and control work needs to be strengthened. Shell, BP, ExxonMobil and other petroleum and petrochemical companies started early in the prevention and control of site contamination, they set up environmental remediation management agencies and established relevant systems (Kovalick and Montgomery, 2017; Bouma and Droogers, 2007; Bluffstone, 2007). China’s petroleum and petrochemical enterprises can learn from the relevant experience of foreign oil companies, and combined with the requirements of domestic laws and regulations, formulate relevant systems for the prevention and control of soil and groundwater contamination, and carry out professional training for relevant personnel to ensure the implementation of the system.

**(2) At the level of local environmental supervision departments and sewage discharge enterprises, strengthen daily environmental supervision, and strengthen the investigation of contamination hidden dangers in key workshops of enterprises in service.**

For the contaminated sites from oil depot of in-service petrochemical enterprises, the investigation of hidden dangers of soil and groundwater contamination can be included in the daily management work, refer to the “Guidelines for the Investigation of Hidden Dangers of Soil Contamination in Key Regulatory Units” regularly carry out hidden danger investigation in key workshops, such as mixed oil treatment area, oily sewage treatment area and hazardous waste temporary storage room, key facilities, such as oil storage tanks, underground pipelines, etc. And strengthen the daily monitoring of soil and groundwater environmental quality in key areas, with reference to the “Technical Guide for Soil and Groundwater self-Monitoring in Industrial Enterprises” to focus on monitoring soil and groundwater in areas with potential contamination risks and around facilities. Timely detection of contamination problems and taking corresponding prevention and control measures can effectively prevent the further expansion of contamination and reduce the contamination risk and treatment costs of enterprises. At the same time, the petroleum and petrochemical enterprises are in the gathering area, so they should do a good job of monitoring at the enterprise boundary and timely find the migration of contaminants to the outside or inside the field through groundwater, so as to reduce the environmental disputes between enterprises.

**(3) At the level of engaged units, increase scientific and technological research and development to solve the problem of control and remediation while producing enterprises in service.**

The prevention and control of soil contamination in China’s petroleum and petrochemical enterprises is still in its infancy. At present, the existing site investigation technology, remediation technology and equipment are rarely used in petroleum and petrochemical enterprises (Lv et al., 2022). And the application of traditional sampling and remediation technology in production enterprises has limitations, which has great disturbance to soil and groundwater, and high requirements for working space and safety in production, so it is difficult to guarantee that it will not interfere with daily production activities. Therefore, we should focus on technical research and development, solve the problems such as disturbance of soil and groundwater in production site, limited clearance of production workshop, and develop drilling sampling and remediation technology suitable for in-service enterprises.

**(4) From the perspective of national double-carbon strategy, optimize green and sustainable technologies to construct the risk prevention and control mode of control and remediation while producing.**

In-service petrochemical enterprises should follow the concept of green development, assume the responsibility for the prevention and control of soil contamination, strengthen the investigation of hidden dangers of contamination and environmental quality monitoring, and do a good job in risk control and remediation of contaminated sites, and provide technical support for the prevention and control of soil and groundwater contamination in-service enterprises, carry out key technologies for risk prevention and control of contaminated sites, and study the risk control and remediation technologies of green, efficient and low-interference soil and groundwater. Construct the risk prevention and control mode of control and remediation while producing.

## 4 Conclusion

The study revealed that soil contamination by heavy metals (Cu, Ni, Cd, Pb, Hg, As) and petroleum hydrocarbons was predominantly localized in the oily sewage treatment area, with vertical migration constrained by silty sand layers. The interaction between surface hydrocarbon accumulation and hydraulic gradient-driven groundwater flow established redox-stratified environments conducive to microbial functional differentiation. Indigenous microbial communities demonstrated robust hydrocarbon-degrading capacities and metabolic plasticity aligned with these redox zones. Specifically, hydrocarbon degradation was driven by Proteobacteria (e.g., unclassified Comamonadaceae; aerobic oxidation), Firmicutes (e.g., Desulfosporosinus; sulfate-coupled anaerobic metabolism), and Basidiomycota (e.g., Trametes; ligninolytic enzymatic hydrolysis) in contamination sources and downstream areas. Critically, fungal-bacterial synergy, which is characterized by fungal peroxidases fragmenting polyaromatics and bacteria mineralizing byproducts, formed a coupled “preprocessing-mineralization” mechanism, underscoring the viability of *in situ* bioremediation anchored to indigenous communities. Furthermore, based on the site’s contamination status and bioremediation feasibility, the study proposes a decision-making framework for contaminated sites from in-service oil depots, emphasizing investigation, monitoring, and remediation strategies. It advocates prioritizing indigenous microbial degradation for long-term risk management, supported by a multi-tiered environmental governance system spanning national policy mandates, subnational regulatory coordination, enterprise-level compliance mechanisms, and strategic alignment with dual-carbon objectives. These findings collectively bridge scientific insights to actionable solutions, offering both technical and policy references for risk management and control in medium-low risk in-service oil depots. However, the findings of this study require further validation across heterogeneous environments. Future research should expand sample coverage, combine multi-site investigations to confirm the universality of microbial metabolic functions, and establish long-term monitoring frameworks to refine risk control strategies under dynamic field conditions. While this study demonstrates the metabolic potential of indigenous microbial communities through taxonomic and function prediction, certain limitations should be noted. Key biodegradation biomarkers (alkB, dsrAB) remain unquantified, and single-point sampling precludes resolution of temporal degradation dynamics. Future studies will utilize qPCR-based quantification of functional biomarkers alongside multi-temporal sampling to refine predictive models for hydrocarbon degradation dynamics, ultimately strengthening bioremediation frameworks under China’s evolving environmental policies.

## Data Availability

The original contributions presented in this study are included in this article/Supplementary material, further inquiries can be directed to the corresponding authors.
