# Supplementary material for: Microbial community dynamics and bioremediation strategies for petroleum contamination in an in-service oil Depot, middle-lower Yellow River Basin
Source: Front Microbiol. 2025 Apr 2;16:1544233. doi: 10.3389/fmicb.2025.1544233 (PMC12000076; doi:10.3389/fmicb.2025.1544233)
Supplement: Supplementary file 1 [file Data_Sheet_1.docx]

**[Supplementary Material](https://www.frontiersin.org/journals/microbiology/articles/10.3389/fmicb.2023.1188229/full#h13)**

**Microbial Community Dynamics and Bioremediation Strategies for Petroleum Contamination in an in-service Oil Depot, Middle-Lower Yellow River Basin**

Quanwei Song^a^, Bingyu Zhou^a,b^, Yinan Song^a^, Xianyuan Du^a^, Hongkun Chen^a^, Rui Zuo^c^, Jin Zheng^a^, Tingyu Yang^b^, Yimin Sang^b*^, Jufeng Li^a**^

^a^CNPC Research Institute of Safety & Environment Technology, Beijing 102206, China;

^b^Department of Environmental Engineering, Beijing Institute of Petrochemical Technology, Beijing 102617, China; ^c^College of Water Sciences, Beijing Normal University, Beijing 100875, China

***Corresponding author.**

****Corresponding author.**

**E-mail:** [**sangyimin@bipt.edu.cn**](mailto:sangyimin@bipt.edu.cn) **(Yimin Sang),** [**ljf69@petrochina.com.cn**](mailto:ljf69@petrochina.com.cn) **(Jufeng Li)**

This [*Supplementary Material*](https://www.frontiersin.org/journals/microbiology/articles/10.3389/fmicb.2023.1188229/full#h13) file has 18 pages including 2 figures and 5 tables.

**Supporting Contents**

**S1 Materials and Methods**

S1.1 The detailed facility layout of the site

S1.2 Identification of suspected contaminated areas and confirmation of distribution areas

S1.3 Illumina sequencing and bioinformatics analysis

**S2 Results and Discussion**

S2.1 Horizontal distribution of soil contamination

S2.2 Vertical distribution of soil contamination

S2.3 Horizontal distribution of groundwater contamination

**Figure Captions**

**Fig. S1** Relative abundance of bacteria and fungi at phylum level in different groundwater samples

**Fig. S2** Distribution of bacterial and fungal phyla present in different groundwater samples

**Table Captions**

**Table S1** Soil and Groundwater detection indicators

**Table S2** Detected contaminant concentrations in soil

**Table S3** Detected contaminant concentrations in groundwater

**Table S4** Sequencing data statistics

**Table S5** The relative abundance data of fungal function prediction inferred by FUNGuild

**References for Supporting Information**

**S1 Materials and Methods**

**S1.1 The detailed facility layout of the site**

Based on the investigation of the enterprise's layout, there are 50,000 cubic meter storage tank area, 20,000 cubic meter storage tank area, oil mixing tank area, process area (including process equipment, oil pump, process valve group), oil mixing treatment area, Xin gang spur line (out of use), firefighting equipment area, oily sewage treatment room, substation, control room (office building) and other areas, most of the ground in the plant has hardening measures. Each oil storage tank area is provided with cofferdam and hardened ground. Among them, the 50,000 m^3^ storage tank area is equipped with 4 steel storage tanks with 50,000 m^3^ internal floating roof, the 20,000 m^3^ storage tank area is equipped with 8 steel storage tanks with 20,000 m^3^ internal floating roof, the oil mixing tank area is equipped with 2 steel storage tanks with 5000m^3^ internal floating roof, and the mixed oil treatment area is equipped with 4 steel storage tanks with 500 m^3^ internal floating roof. This station has the storage capacity of 370,000 m^3^ of refined oil, and has the functions of pigging, filtering, metering, distribution, pressurization, mixed oil treatment and oil storage.

**S1.2 Identification of suspected contaminated areas and confirmation of distribution areas**

According to the sequence of suspected contaminated areas given in the "Technical Provisions for the Layout of Suspected Polluted Plots in Land Use Investigations for Key Industry Enterprises (Trial)" and the key focus areas of in-service enterprises, the suspected contaminated areas of this site are identified, and the final distribution areas is determined based on the screening and layout principles.

1. **Areas where leakage or environmental contamination accidents have occurred**

According to the previous site survey and data analysis, the site was farmland before construction, and there was no contamination from historical industrial enterprises, and through data collection, on-site inspection and personnel interviews, we know that no contamination incident was found in this oil transportation station. Therefore, this type of suspected contaminated area does not exist.

1. **Areas where underground pipelines are located**

The whole line of this operation area adopts airtight transportation technology, and it is buried deep underground. Under the normal operating conditions during the operation period, the main pipeline does not produce and discharge contaminants. Therefore, this area does not serve as the distribution area for this survey.

1. **Areas for storage, loading, unloading, use and disposal of raw and auxiliary materials**

The main purpose of the site is to transport and store gasoline and diesel oil. The site mainly uses above-ground storage tanks as storage facilities and underground oil pipelines as oil transportation facilities. Through investigation, it is believed that the gasoline and diesel oil storage tank area is suspected of contamination. In addition, the main process of the site also includes the oil mixing treatment process of raw oil, which involves the oil mixing storage tank area and the oil mixing treatment area. However, due to the actual geographical location of the two areas and the same contaminants type, only the oil mixing treatment area can be considered. Therefore, the 20,000 cubic tank area, the 50,000 cubic tank area and the mixed oil treatment area are used as the distribution areas of this investigation.

1. **Areas of enterprise contamination prevention and control facilities**

The air contaminants in the station mainly include the breathing exhaust gas of the storage tank size, the reboiler furnace and the gas boiler exhaust gas from the station, by adopting the internal floating roof tank and the exhaust cylinder of a certain height, the emission of unorganized exhaust gas can be reduced. The solid waste generated by the site mainly includes waste residue at the bottom of oil tanks, oily rags, filter cleaning oil residue and household waste, etc. The site shall collect, store, transport and dispose of hazardous waste in strict accordance with the relevant provisions of hazardous waste, and then entrust qualified units to deal with it; domestic waste is mainly treated in a unified manner through centralized collection, regular sanitation clearance and transportation, and solid waste is collected and stored in solid waste temporary storage rooms. The waste water in the station is mainly oily sewage generated by inspection and maintenance and domestic sewage of the staff. For oily wastewater generated by inspection and maintenance, oily wastewater treatment devices are set up in the field for treatment, and the effluent is temporarily stored in the oily sewage treatment tank for cleaning operations during storage tank overhaul; after the domestic sewage is treated by the construction of the buried integrated domestic sewage treatment device, it is used for the greening of the factory area and is not discharged. Since solid waste and oily sewage may cause site contamination, considering the actual situation and the close location of the two areas, considering the project workload arrangement, the oily sewage treatment area is selected as the distribution area of this investigation.

After identifying the suspected contaminated areas of the site, according to the principle of screening the distribution, the distribution area is finally determined to be 20,000 cubic meters storage tank area, 50,000 cubic meters storage tank area, mixed oil treatment area and oily sewage treatment area.

**S1.3 Illumina sequencing and bioinformatics analysis**

**1.3.1 Sequence Quality Control**

①Initial QC with SMURF 5R: Raw sequencing data were pre-processed using SMURF 5R software (in-built quality control module) to remove low-complexity sequences and potential contaminants.

②Secondary QC with fastp: Further trimming of low-quality bases (Phred score <20) and adapter removal were performed using fastp v0.19.6.

**1.3.2 PCR Amplification**

**①Primer Sequences:**

Bacterial 16S rRNA V3–V4 region:

338F:5′-ACTCCTACGGGAGGCAGCAG-3′

806R:5′-GGACTACHVGGGTWTCTAAT-3′

Fungal ITS region:

ITS1F:5′-CTTGGTCATTTAGAGGAAGTAA-3′

ITS2R:5′-GCTGCGTTCTTCATCGATGC-3′

**②Reaction Setup (20 μL):**

2× TransStart FastPfu Master Mix: 10 μL

Forward Primer (5 μM): 0.8 μL

Reverse Primer (5 μM): 0.8 μL

DNA Template (10 ng/μL): 2 μL

ddH₂O to 20 μL.

**③Thermal Cycler Conditions (ABI GeneAmp® 9700):**

Bacterial samples:

Denaturation: 95°C, 3 min

Cycling (29 cycles): 95°C 30 s → 53°C 30 s → 72°C 45 s

Final extension: 72°C, 10 min

Fungal samples:

Denaturation: 95°C, 3 min

Cycling (35 cycles): 95°C 30 s → 55°C 30 s → 72°C 45 s

Final extension: 72°C, 10 min

**④Amplification Validation:**

Electrophoresis: 2% agarose gel (100 V, 30 min) with DL2000 DNA Marker.

Result Criteria:

Grade A: Correct band size (bacteria: ~468 bp; fungi: ~350 bp) and optimal concentration.

Grade B/C: Re-amplification required.

**S2 Results and Discussion**

**S2.1** **Horizontal distribution of soil contamination**

In order to compare and explore the soil contamination status at different points of the site, the concentration values of all detected indicators at T1~T6 points at each sampling depth were averaged, and the horizontal distribution of soil contamination status at the site were analyzed by drawing.

1. **Heavy metals**

The mass concentration distribution of heavy metals such as copper, nickel, cadmium, lead, mercury and arsenic at T1~T6 points is shown in Fig. 3a.

It can be seen from Fig. 3a that 6 kinds of heavy metals were detected at T1~T6 points, and the contamination concentrations of all heavy metals are far lower than the screening values of the first and second types of land for "Soil Environmental Quality Construction Land Risk Control Standard (trial)" (GB 36600-2018). The concentrations of copper, nickel, cadmium, mercury and arsenic at different sampling points had little difference, and there was no obvious division of high and low values. However, for lead, it was found that the concentration of contamination at T1 was significantly higher than that at the other five points. T1 was located in the south of the 20,000 m3 oil tank area, which was mainly responsible for the storage of gasoline. By comparing with T2 in the same area of gasoline tank area, the possibility of failure or aging leakage of storage equipment in the oil tank area is ruled out, and combined with the historical use of the station plot, no additional lead contamination sources are found at T1 compared with other points. Therefore, it was believed that the higher lead concentration at T1 compared with other points in this survey is caused by other human sources.

The organic contaminants at T1~T6 points was detected, among which petroleum hydrocarbons were only detected at T5 (15.0 mg/kg), and other organic contaminants were detected as shown in Fig. 3b.

It can be seen from Fig. 3b that only chloromethane, dichloromethane, tetrachloroethylene and chloroform can be detected. Among them, chloromethane was detected in all 6 points, and the concentration was in the order of 10-3, which was 1/10000 of the screening value of the first category of "Soil environmental quality-Risk control standard for soil contanination of development land" (GB 36600-2018), dichloromethane was detected in T1, T2, T3 and T6, and tetrachloroethylene was detected in T4, T5 and T6, and their content in soil was only 1/10000 of the selected value. Chloroform was detected in all T1~T6 sampling points, and the detected concentrations were of the order of 10-3, which was only 1% of the screening value of the first kind of land in "Soil environmental quality-Risk control standard for soil contanination of development land" (GB 36600-2018). Overall, the level of soil contamination is low.

**S2.2** **Vertical distribution of soil contamination**

Soil samples were collected from T1~T6 points in the range of 0~5 m, 0.5~3 m and 3~6 m, and the vertical distribution of contaminants detected in the soil survey were analyzed.

1. **pH value**

Soil pH value is one of the important indicators to reflect the physical and chemical properties of soil, which not only controls the existing form and bioavailability of metal elements and many other physical and chemical processes ([Xu et al., 2020](#_ENREF_60)), but also has an important impact on the activities of bacteria and fungi in soil ([Aziz et al., 2024](#_ENREF_4)). Therefore, exploring the spatial distribution of soil pH is helpful to clarify the migration process of contaminants in the site, and can provide a basis for the use of soil microorganisms for risk control of remediation.

The result of pH change is shown in Fig. 4a. From the surface layer to the middle layer of the soil, the soil pH value at all points increases significantly, while from the middle layer to the deep layer, the pH value basically does not change. It shows that the soil alkalinity of the site from oil depot increases with the increase of soil depth, but when it reaches a certain depth, the value is basically stable, and the pH value of all points is about 8.3.

1. **Heavy metals**

The vertical distribution of heavy metals is shown in Fig. 4b. First of all, on the whole, it is found that most of the detected heavy metals show the trend of "surface enrichment". The reason for this is roughly due to the distribution of soil texture and soil organic matter.

According to the results of soil drilling in the site, the texture of surface and middle soil was silt (silt is between sand and clayey soil, the content of particles larger than 0.075mm is not more than 50% of total weight), and the deep soil texture is silt (the content of particles larger than 0.075mm is more than 50% of total weight), indicating that the particle size of surface and middle soil was relatively small. The smaller the soil particle size and the larger the specific surface area, the stronger the adsorption capacity of heavy metals, that is, the more difficult the migration of heavy metal ions in the soil, heavy metals are easy to enrich in the surface and middle soil. Considering that the input of foreign organic matter is easy to be obtained in the surface soil, the content of organic matter in the surface soil is significantly higher than that in the lower layer, and the main component of soil organic matter is humus, which has a strong complexation and adsorption effect on heavy metals. Therefore, the surface soil with more organic matter has stronger adsorption and complexation ability to heavy metal ions ([Yang et al., 2013](#_ENREF_64)). Therefore, the heavy metals in the soil of the site should be more easily distributed in the soil layer closer to the ground.

Secondly, it can be found that individual points do not conform to the overall surface enrichment trend, but show other characteristic trends.

①The mass concentration of Cu at T2, T4 and T5 points, Ni at T2 and T4 points, Hg at T5 points and As at T1 and T5 points all decreased first and then increased with the deepening of soil depth, and even the concentration of deep soil at individual points exceeded that of surface soil. The soil mass concentration in the surface layer was higher than that in the middle layer, which could be inferred to be caused by the contamination in the functional area of the site. In view of the fact that the area around the site before construction and the site are farmland, copper, nickel, mercury and arsenic are elements contained in chemical fertilizer, and excessive use of chemical fertilizer will lead to an increase in the content of heavy metal elements in soil. Therefore, it is believed that the increase in the content of copper, nickel, mercury and arsenic in the deep soil at the above sites is due to the accumulation of downward migration of historical contamination sources under the action of rainfall leaching; ②The mass concentration of Ni at T3 and Hg at T3 increased first and then decreased rapidly with the increase of soil depth. It is speculated that the anti-seepage materials at T3 have aging or damage problems, so they can not effectively block the infiltration of heavy metal contamination, and the soil is contaminated. Over time, the nickel and mercury in the surface soil will migrate downward with water. Because the permeability of the middle soil is worse than that of the deep soil, the final enrichment is in the middle depth layer of 0.5~3.0 m; ③The mass concentration of Pb at T3 and As at T4 showed a trend of gradual increase with the increase of soil depth. It is believed that when the historical land was used as farmland, the abuse of pesticides (lead being the main active component) and fertilizers (containing arsenic) caused some soil contamination, and then migrated downward due to rain leaching, and finally enriched in deep soil; ④The mass concentration of Hg at T1, T2 and T4 first decreased with the increase of soil depth, and then the concentration basically remained stable and no longer changed with the depth. The mass concentration in the surface soil was significantly higher than that in the middle and deep soil, which was considered to be caused by site contamination in the surface soil, while the concentration in the deep soil did not decrease significantly compared with that in the middle layer, and the concentration basically stabilized, which could be considered to be less affected by contamination, and the contamination content in the middle and deep soil remained at the background level of mercury in the area.

Based on the contamination distribution and source analysis of the above 6 detected heavy metals, it was found that except for some points where the concentration of heavy metals in deep soil was high due to the historical use of the land and the surrounding environment, the heavy metals in most points showed a trend of gradually decreasing with the increase of soil depth. It was also found that at point T5, the concentrations of the 6 detected heavy metals in the surface soil were higher than those in the middle and deep soils. Therefore, it was believed that during the operation of this station, this point was contaminated to a certain extent. Combined with the fact that petroleum hydrocarbons were only detected at T5, it can be determined that point T5 was contaminated by oil caused by accidental dripping of the oily wastewater treatment device or the accumulation of uncollected oily waste residues (waste residues at the bottom of oil tanks, oily rags, filter cleaning oil residues, etc.). In addition, based on the distribution results of nickel and mercury at T3, it can be concluded that the anti-seepage material at this point may have aging problems.

1. **Organic contaminants**

The vertical distribution of organic matter is shown in Fig. 4c. Petroleum hydrocarbons were detected only in the surface soil at T5 point, and the mass concentrations in the middle and deep layers were lower than the detection limits, which further proved that petroleum hydrocarbons detected at T5 point were caused by oil contamination at the site from oil depot, rather than historical contamination sources. The other 4 organic contaminants were VOCs. First of all, in general, the organic contaminants in most soil points show a trend of "surface enrichment", that is, from the surface to the middle soil, the mass concentration decreases significantly, while from the middle soil to the deep soil, the mass concentration is basically unchanged.

Secondly, the vertical distribution of volatile organic compounds in each point was observed respectively. It can be found that at points T1, T2, T3, T4, and T6, as long as chloroform, dichloromethane, or tetrachloroethylene was detected, the contaminant concentration decreased first and then stabilizes with the increase of soil depth. Since T1 and T2 were located in the 20,000 cubic meter oil tank area, T3 and T4 were located in the mixed oil treatment area, and T6 was located in the 50,000 cubic meter oil tank area, it was believed that VOCs are mainly produced by oil tank breathing and heating furnace flue gas emissions. After entering the atmosphere as volatile gases, they were adsorbed on particulate matter and eventually contaminate the surface soil through atmospheric dust precipitation. There was basically no difference in the vertical distribution of chloroform content in points T1~T4, and there was no obvious contamination enrichment in the surface soil, indicating that these four points were not contaminated by station operations. The detected chloroform mainly comes from natural sources, which further proves the previous speculation. At point T6, there was an obvious surface enrichment phenomenon. It was speculated that the chloroform is produced by the oil storage tanks in this area due to tank breathing; for point T5 located in the northwest corner of the site, due to the northwest wind during the sampling period, as the upwind direction, point T5 was less contaminated by the atmosphere, and the vertical distribution of methyl chloride, dichloromethane, tetrachloroethylene and chloroform has basically not changed.

**S2.3** **Horizontal distribution of groundwater contamination**

In order to compare and explore the groundwater contamination status at different points of the site, the concentration values of heavy metals, petroleum hydrocarbons and trichloromethane detected by monitoring wells D1~D6 were mapped to analyze the horizontal distribution characteristics of groundwater contamination status at the site.

1. **Heavy metals**

It can be seen from Fig. 5a that among the five detected heavy metals, the contents of iron, manganese and aluminum in each monitoring well are all lower than the Class II standard limit in the "Standard for groundwater quality" (GB/T14848-2017); The detected concentrations of arsenic and chromium in D1~D6 monitoring wells were higher than the standard limits of Class II groundwater, and the detected concentrations of these two heavy metals were significantly lower at D6 and similar in D1~D5. It is believed that this phenomenon is due to the fact that the D6 monitoring well is located in the northwest corner of the site, while the groundwater of the plant flows from northwest to southeast. The D6 is located in the upper reaches of the groundwater, and the contamination spreads downstream through the groundwater flow, so the contamination concentration of the D6 monitoring well is relatively low.

1. **Organic contaminants**

As can be seen from Fig. 5b, petroleum hydrocarbons were detected in all D1~D6 monitoring wells. Since almost all groundwater monitoring points were common to soil and water, and petroleum hydrocarbons in soil were only detected at T5 (corresponding to wells in D6 oily wastewater treatment area, northwest corner of the site), combined with the flow direction of groundwater, it can be considered that the petroleum hydrocarbons detected in groundwater in D6 are derived from soil leaching, while the contamination of petroleum hydrocarbons in the other five places is caused by groundwater flow. However, it is found that the concentration of petroleum hydrocarbons in the contamination source D6 monitoring well is lower than that in the downstream monitoring well D5, which is the same as the horizontal distribution of arsenic and chromium. Combined with the analysis of the horizontal distribution of heavy metals, it can be comprehensively concluded that T5 (oily sewage treatment area and hazardous waste temporary storage room) has had problems such as accidental dripping of oily sewage or accumulation of oil residue, which caused oil contamination at D6 by leaching. and with the flow of groundwater leads to the enrichment of contaminants downstream. The concentrations of chloroform detected in D1~D6 monitoring wells are only slightly different, and are lower than the standard limits of type Ⅱ and type Ⅲ groundwater. Furthermore, because chloroform has high leaching mobility ([Ya-song et al., 2011](#_ENREF_63)), it is very easy to use soil as a migration channel to enter groundwater under the action of rain water, so it can be considered that chloroform in groundwater is mainly caused by trichloromethane leaching from natural sources in soil.

**Tables**

**Table S1** Soil and Groundwater detection indicators

| **Monitoring Target** | **Detection Indicators** |
| --- | --- |
| Soil | 45 basic items (including 7 heavy metals and inorganics, 27 volatile organics, 11 semi-volatile organics), pH value, total petroleum hydrocarbons (C10-C40), totaling 47 items |
| Groundwater | Color, odor and taste, turbidity, visible matter to the naked eye, pH, total hardness, dissolved total solids, total sulfates, chlorides, iron, manganese, copper, zinc, aluminum, volatile phenols, anionic surfactants, Chemical Oxygen Demand (COD), ammoniacal nitrogen, sulfides, sodium, total coliform bacteria, total bacteria count, nitrite, nitrates, cyanides, fluorides, iodide, mercury, arsenic compounds, selenium, lead, chloroform, carbon tetrachloride, benzene, toluene, total α radioactivity, total β radioactivity, total petroleum hydrocarbons (C6-C9 and C10-C40), ethylbenzene, ortho-xylene, meta-para-xylene, methyl tert-butyl ether, totaling 44 items |

| Sampling point | Sampling depth | Cu  (mg/kg) | Ni  (mg/kg) | C10-C40 (mg/kg) | pH  (无量纲) | Cd  (mg/kg) | Pb  (mg/kg) | Hg  (mg/kg) | As  (mg/kg) | Chloromethane  (mg/kg) | Dichloromethane  (mg/kg) | Tetrachloroethylene  (mg/kg) | Chloroform  (mg/kg) |
| --- | --- | --- | --- | --- | --- | --- | --- | --- | --- | --- | --- | --- | --- |
| **T1** | 0-50 (cm) | 17.3 | 20 | ND | 8.33 | 0.05 | 34.6 | 0.024 | 8.62 | 1.9×10^-3^ | 9.6×10^-3^ | / | 3.8×10^-3^ |
|  | 50-300 (cm) | 16.3 | 19 | ND | 8.28 | 0.05 | 32.6 | 0.013 | 3.65 | 1.4×10^-3^ | ND | / | 2.7×10^-3^ |
|  | 300-600 (cm) | 15.0 | 20 | ND | 8.25 | 0.04 | 20.8 | 0.010 | 8.33 | 1.3×10^-3^ | ND | / | ND |
| **T2** | 0-50 (cm) | 15.800 | 17.000 | ND | 8.200 | 0.050 | 21.300 | 0.015 | 8.730 | 1.8×10^-3^ | 0.016 | / | 3.3×10^-3^ |
|  | 50-300 (cm) | 12.8 | 16 | ND | 8.65 | 0.04 | 18.5 | 9×10^-3^ | 7.62 | 1.4×10^-3^ | 2.5×10^-3^ | / | 2.9×10^-3^ |
|  | 300-600 (cm) | 17.8 | 21 | ND | 8.33 | 0.05 | 19.5 | 9×10^-3^ | 4.26 | 1.3×10^-3^ | ND | / | 2.3×10^-3^ |
| **T3** | 0-50 (cm) | 16.4 | 19 | ND | 8.28 | 0.05 | 19.9 | 0.022 | 8.00 | 1.6×10^-3^ | 3.7×10^-3^ | ND | 7.6×10^-3^ |
|  | 50-300 (cm) | 15.4 | 23 | ND | 8.30 | 0.05 | 22.2 | 0.036 | 8.21 | 1.2×10^-3^ | ND | ND | 3.4×10^-3^ |
|  | 300-600 (cm) | 11.7 | 17 | ND | 8.47 | 0.04 | 23.6 | 0.012 | 7.66 | 1.1×10^-3^ | ND | ND | ND |
| **T4** | 0-50 (cm) | 15.0 | 18 | ND | 7.99 | 0.05 | 22.2 | 0.030 | 8.18 | 2.0×10^-3^ | ND | 9.1×10^-3^ | 5.3×10^-3^ |
|  | 50-300 (cm) | 13.1 | 13 | ND | 8.27 | 0.04 | 18.5 | 0.011 | 8.53 | 1.5×10^-3^ | ND | ND | 2.7×10^-3^ |
|  | 300-600 (cm) | 14.6 | 19 | ND | 8.29 | 0.04 | 19.6 | 0.011 | 11.4 | 1.5×10^-3^ | ND | ND | ND |
| **T5** | 0-50 (cm) | 16.4 | 20 | 15 | 8.13 | 0.07 | 23.8 | 0.022 | 11.0 | 1.2×10^-3^ | ND | 1.6×10^-3^ | 2.8×10^-3^ |
|  | 50-300 (cm) | 13.6 | 18 | ND | 8.33 | 0.04 | 20.4 | 0.014 | 5.42 | 1.2×10^-3^ | ND | 1.8×10^-3^ | 3.6×10^-3^ |
|  | 300-600 (cm) | 14.6 | 17 | ND | 8.37 | 0.03 | 19.5 | 0.018 | 7.94 | 1.2×10^-3^ | ND | ND | 2.7×10^-3^ |
| **T6** | 0-50 (cm) | 16.7 | 21 | ND | 8.24 | 0.05 | 18.8 | 0.034 | 9.12 | 1.5×10^-3^ | 1.9×10^-3^ | 4.1×10^-3^ | 0.0219 |
|  | 50-300 (cm) | 15.7 | 20 | ND | 8.30 | 0.05 | 18.1 | 0.020 | 7.37 | 1.1×10^-3^ | 2.1×10^-3^ | 2.2×10^-3^ | 2.3×10^-3^ |
|  | 300-600 (cm) | 14.4 | 21 | ND | 8.30 | 0.04 | 18.3 | 8×10^-3^ | 3.76 | 1.2×10^-3^ | ND | 2.7×10^-3^ | 2.4×10^-3^ |

**Table S2** Detected contaminant concentrations in soil

**Table S3** Detected contaminant concentrations in groundwater

| **Detection indicator** | | **Detection limit** | **Monitoring well** | | | | | | **Evaluation standard** | **Standard source** |
| --- | --- | --- | --- | --- | --- | --- | --- | --- | --- | --- |
|  |  |  | **D1** | **D2** | **D3** | **D4** | **D5** | **D6** |  |  |
| Color | | 5 | 10 | 10 | 10 | 10 | 10 | 10 | ≤15 | the Class III water quality standard of "Standard for Groundwater Quality" (GB/T14848-2017) |
| Odor and Taste | | / | / | / | / | / | / | / | / |  |
| Turbidity (NTU) | | 0.3 | 2.8 | 2.7 | 2.6 | 2.8 | 2.5 | 2.6 | ≤3 |  |
| Visible Particulates | | / | / | / | / | / | / | / | / |  |
| pH | | / | 7.2 | 7.1 | 6.9 | 7.2 | 7.1 | 7.1 | 6.5≤pH≤8.5 |  |
| Total Hardness (mg/L) | | / | 373 | 252 | 234 | 385 | 389 | 240 | ≤450 |  |
| Dissolved Total Solids (mg/L) | | 10 | 671 | 339 | 722 | 612 | 489 | 357 | ≤1000 |  |
| Sulfates (mg/L) | | 0.018 | 82.8 | 58.3 | 74.6 | 89.4 | 83.2 | 66.8 | ≤250 |  |
| Chlorides (mg/L) | | 7×10-3 | 19.8 | 14.7 | 15.0 | 20.0 | 20.3 | 14.0 | ≤250 |  |
| Fe (mg/L) | | 0.03 | ND | 0.04 | 0.06 | ND | ND | 0.04 | ≤0.3 |  |
| Mn (mg/L) | | 0.01 | ND | ND | 0.03 | ND | ND | ND | ≤0.1 |  |
| Cu (mg/L) | | 0.01 | ND | ND | ND | ND | ND | ND | ≤1.00 |  |
| Zn(mg/L) | | 0.01 | ND | ND | ND | ND | ND | ND | ≤1.00 |  |
| Al (μg/L) | | 1.15 | 3.73 | 3.07 | 3.42 | 3.63 | 4.45 | 3.95 | ≤200 |  |
| Volatile Phenols (mg/L) | | 3×10-4 | 1.4×10-3 | 1.7×10-3 | 1.5×10-3 | 1.1×10-3 | 1.8×10-3 | 1.4×10-3 | ≤0.002 |  |
| Anionic Surfactants (mg/L) | | 0.05 | ND | ND | ND | ND | ND | ND | ≤0.3 |  |
| COD (mg/L) | | 0.05 | 2.58 | 2.51 | 2.17 | 1.95 | 2.12 | 1.66 | ≤3.0 |  |
| Ammoniacal Nitrogen (mg/L) | | 0.025 | 0.448 | 0.469 | 0.463 | 0.469 | 0.472 | 0.480 | ≤0.50 |  |
| Sulfides (mg/L) | | 3×10-3 | ND | ND | ND | ND | ND | ND | ≤0.02 |  |
| Sodium (mg/L) | | 0.01 | 14.7 | 10.2 | 14.6 | 14.1 | 16.2 | 7.83 | ≤200 |  |
| Total Coliform Bacteria (MPN/100mL) | | / | ND | ND | ND | ND | ND | ND | ≤3.0 |  |
| Total Bacteria Count (CFU/mL) | | / | 13 | 2 | 5 | 9 | 15 | 7 | ≤100 |  |
| Nitrite (mg/L) | | 5×10-3 | ND | ND | 0.660 | ND | ND | ND | ≤1.00 |  |
| Nitrates (mg/L) | | 4×10-3 | 12.8 | 7.49 | 13.1 | 13.6 | 13.4 | 6.81 | ≤20.0 |  |
| Cyanides (mg/L) | | 2×10-3 | ND | ND | ND | ND | ND | ND | ≤0.05 |  |
| Fluorides (mg/L) | | 6×10-3 | 0.334 | 0.217 | 0.562 | 0.343 | 0.332 | 0.216 | ≤1.0 |  |
| Iodide (mg/L) | | 0.05 | ND | ND | ND | ND | ND | ND | ≤0.08 |  |
| Hg (μg/L) | | 0.04 | ND | ND | ND | ND | ND | ND | ≤1 |  |
| As (μg/L) | | 0.3 | 1.3 | 1.3 | 1.4 | 1.3 | 1.5 | 1.1 | ≤10 |  |
| Se (μg/L) | | 0.4 | ND | ND | ND | ND | ND | ND | ≤010 |  |
| Cd (μg/L) | | 1.0 | ND | ND | ND | ND | ND | ND | ≤5 |  |
| Cr (mg/L) | | 4×10-3 | 0.026 | 0.025 | 0.026 | 0.027 | 0.027 | 0.021 | ≤0.05 |  |
| Pb (μg/L) | | 2.50 | ND | ND | ND | ND | ND | ND | ≤10 |  |
| Chloroform (μg/L) | | 1.4 | 2.2 | 2.2 | 1.8 | 2.0 | 1.5 | 2.4 | ≤60 |  |
| Carbon Tetrachloride (ug/L) | | 1.5 | ND | ND | ND | ND | ND | ND | ≤2.0 |  |
| Benzene (μg/L) | | 1.4 | ND | ND | ND | ND | ND | ND | ≤10.0 |  |
| Toluene (μg/L) | | 1.4 | ND | ND | ND | ND | ND | ND | ≤700 |  |
| Total α Radioactivity (Bg/L) | | 0.043 | 0.165 | 0.181 | 0.170 | 0.195 | 0.162 | 0.138 | ≤0.5 |  |
| Total β Radioactivity (Bq/L) | | 0.015 | 0.141 | 0.150 | 0.190 | 0.131 | 0.101 | 0.106 | ≤1.0 |  |
| Ethylbenzene (μg/L) | | 0.8 | ND | ND | ND | ND | ND | ND | ≤300 |  |
| Xylene (total amount) (μg/L) | Ortho-Xylene | 1.4 | ND | ND | ND | ND | ND | ND | ≤500 |  |
|  | Meta-Para-Xylene | 2.2 | ND | ND | ND | ND | ND | ND |  |  |
| Methyl Tert-Butyl Ether (μg/L) | | 0.5 | ND | ND | ND | ND | ND | ND | ≤20 | the "U.S. EPA Drinking Water Health Advisories" |
| TPH (mg/L) (C6-C9 and C10-C40) | | 0.01 | 0.02 | 0.04 | 0.03 | 0.04 | 0.04 | 0.03 | <0.05 | the "Dutch soil and groundwater Environmental Standard" (2013) |

**Table S4** Sequencing data statistics

| Sample | Sequencing Primer | Raw Reads | High-quality Reads | ASV | Total Bases (bp) | Mean Length (bp) |
| --- | --- | --- | --- | --- | --- | --- |
| BD5 | 338F_806R | 168,100 | 64,472 | 1,166 | 37,958,482 | 424.28 |
| BD6 |  |  | 61,587 | 949 | 30,419,962 | 422.78 |
| FD5 | ITS1F_ITS2R | 162,304 | 97,819 | 119 | 23,462,794 | 236.72 |
| FD6 |  |  | 58,913 | 258 | 14,999,811 | 251.93 |

Bacterial (BD5, BD6) and fungal (FD5, FD6) samples were sequenced in paired-end mode (PE300), generating total single-end reads: 168,100 and 162,304, respectively. Post-QC clean reads and sequence lengths are summarized in Supplementary Table S4.

**Table S5** The relative abundance data of fungal function prediction inferred by FUNGuild

| Guild | FD5 | FD6 |
| --- | --- | --- |
| Animal Endosymbiont-Animal Pathogen-Endophyte-Plant Pathogen-Undefined Saprotroph | 339 | 0 |
| Animal Pathogen | 278 | 611 |
| Animal Pathogen-Dung Saprotroph-Endophyte-Epiphyte-Plant Saprotroph-Wood Saprotroph | 57 | 3281 |
| Animal Pathogen-Dung Saprotroph-Endophyte-Lichen Parasite-Plant Pathogen-Undefined Saprotroph | 31 | 0 |
| Animal Pathogen-Endophyte-Epiphyte-Plant Pathogen-Undefined Saprotroph | 103 | 80 |
| Animal Pathogen-Endophyte-Ericoid Mycorrhizal-Plant Pathogen-Wood-Saprotroph | 16 | 152 |
| Animal Pathogen-Endophyte-Fungal Parasite-Plant Pathogen-Wood Saprotroph | 22 | 0 |
| Animal Pathogen-Endophyte-Fungal Parasite-Plant Pathogen-Wood Saprotroph | 7 | 127 |
| Animal Pathogen-Endophyte-Lichen Parasite-Plant Pathogen-Soil Saprotroph-Wood Saprotroph | 155 | 0 |
| Animal Pathogen-Endophyte-Lichen Parasite-Plant Pathogen-Wood Saprotroph | 2234 | 3462 |
| Animal Pathogen-Endophyte-Plant Pathogen-Undefined Saprotroph | 0 | 213 |
| Animal Pathogen-Endophyte-Plant Pathogen-Wood Saprotroph | 2088 | 339 |
| Animal Pathogen-Plant Pathogen-Undefined Saprotroph | 1664 | 1642 |
| Animal Pathogen-Soil Saprotroph | 4562 | 22018 |
| Animal Pathogen-Undefined Saprotroph | 93 | 273 |
| Dung Saprotroph-Endophyte-Wood Saprotroph | 94 | 0 |
| Dung Saprotroph-Plant Saprotroph | 70 | 0 |
| Dung Saprotroph Plant Saprotroph-Wood Saprotroph | 214 | 208 |
| Dung Saprotroph-Soil Saprotroph | 242 | 10796 |
| Endophyte | 60 | 0 |
| Endophyte-Lichen Parasite-Plant Pathogen-Undefined Saprotroph | 397 | 0 |
| Endophyte-Litter Saprotroph-Soil Saprotroph-Undefined Saprotroph | 22298 | 2400 |
| Endophyte-Plant PathogenUndefinedSaprotroph | 13 | 0 |
| Endophyte-Plant Pathogen-Wood Saprotroph | 109 | 0 |
| Epiphyte | 27 | 0 |
| Fungal Parasite | 406 | 0 |
| Fungal Parasite-Litter Saprotroph | 149 | 364 |
| Fungal Parasite-Plant Pathogen-Plant Saprotroph | 0 | 375 |
| Fungal Parasite-Plant Pathogen-Undefined Saprotroph | 32 | 0 |
| Fungal Parasite-Undefined Saprotroph | 229 | 0 |
| Lichenized | 9 | 0 |
| Plant Pathogen | 9934 | 756 |
| Plant Pathogen-Plant Saprotroph | 243 | 1308 |
| Plant Pathogen-Wood Saprotroph | 15 | 239 |
| Soil Saprotroph | 74 | 317 |
| Undefined Saprotroph | 8362 | 6350 |
| Wood Saprotroph | 304 | 805 |
| unknown | 3983 | 2797 |

**Figures**


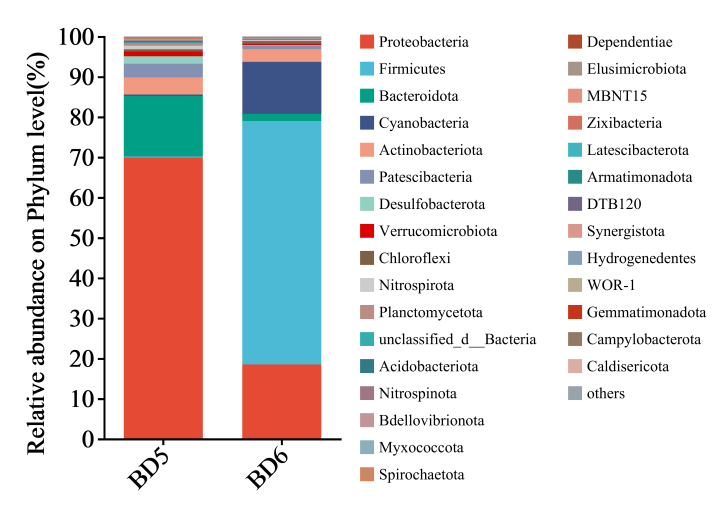

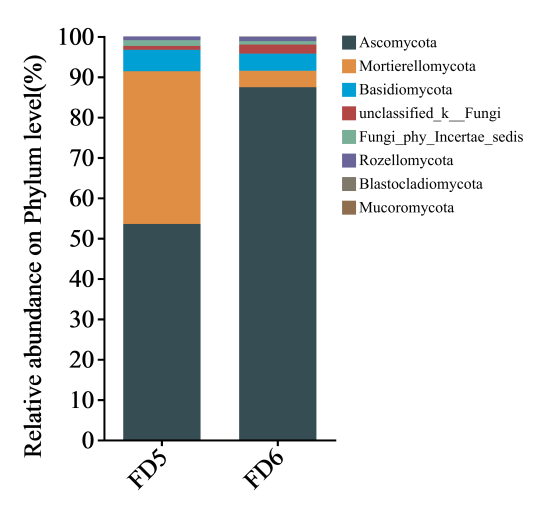


(a) Bacteria (b) Fungi

**Fig. S1** Relative abundance of bacteria and fungi at phylum level in different groundwater samples


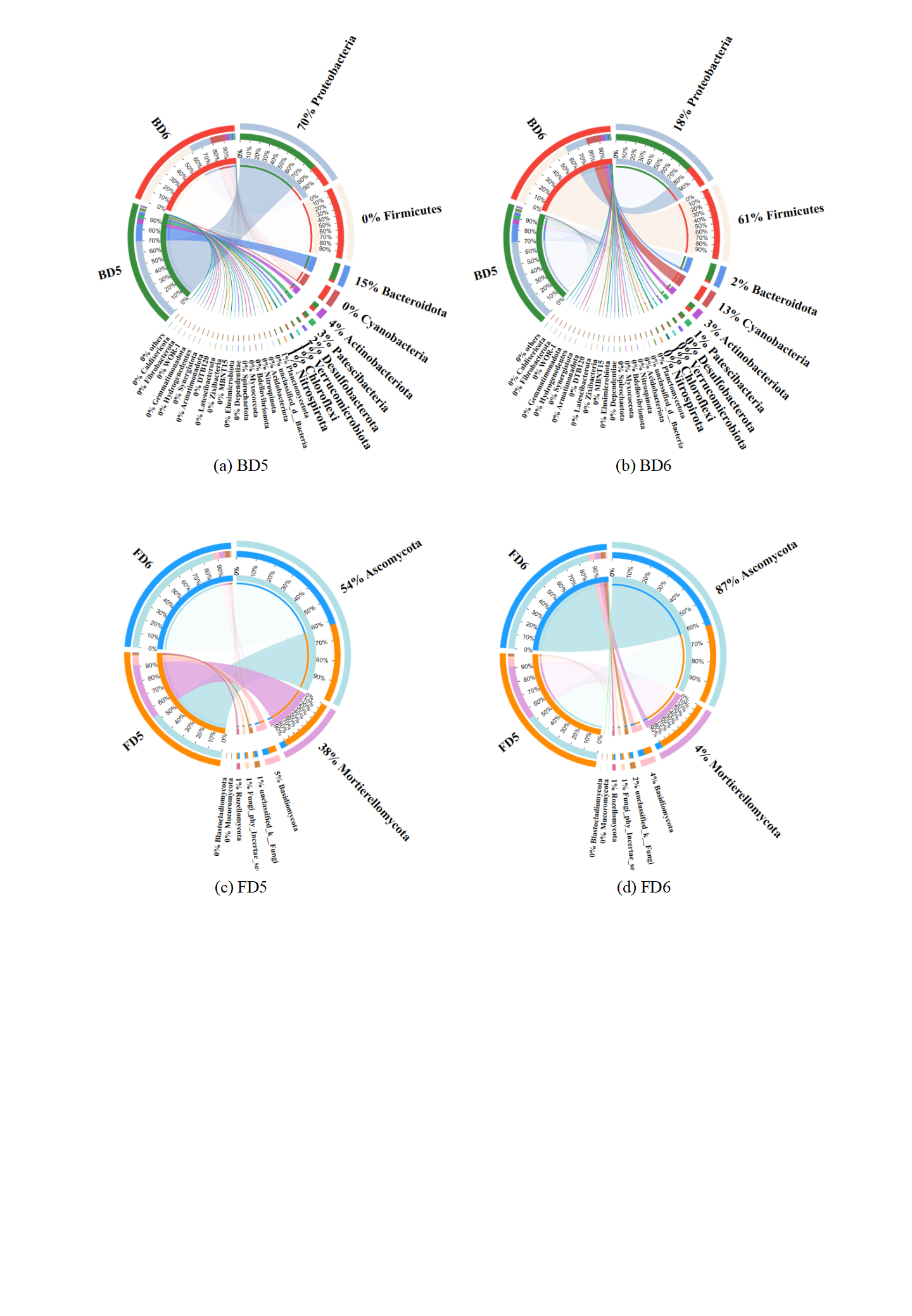


**Fig. S2** Distribution of bacterial and fungal phyla present in different groundwater samples
